# Supplementary material for: Genome-wide detection of human 5′ UTR variants that impact protein translation
Source: Am J Hum Genet. 2026 Mar 24;113(4):809–27. doi: 10.1016/j.ajhg.2026.02.020 (PMC13087467; doi:10.1016/j.ajhg.2026.02.020)
Supplement: Document S1. Figures S1–S7 [file mmc1.pdf]

The American Journal of Human Genetics, Volume *113*

Supplemental information

Genome-wide detection of human 5′ UTR variants that impact protein translation

Matthieu Chaldebas, Khoren Ponsin, Jonathan Bohlen, Clement Conil, Haralambos Mourelatos, Peter D. Stenson, David N. Cooper, Laurent Abel, Jean-Laurent Casanova, Aurélie Cobat, and Peng Zhang

Supplemental Figures

Figure S1: Comparison of WES and WGS coverage on MANE transcript 5' UTR intervals. Distribution of per-sample coverage metrics for 15,680 whole-exome sequencing (WES) and 1,721 whole-genome sequencing (WGS) samples for our in-house database. Each panel displays violin plots showing distribution density, overlaid box plots depicting the median (center line), interquartile range (IQR; box edges), and whiskers extending to 1.5 times the IQR (or the most extreme data point within that range). (a) Median sequencing coverage per sample calculated across 5'UTRs (y-axis on a $\log_{10}$ scale). (b) Distribution of the percentage of the 5'UTR covered at a depth of at least 10X. (c) Distribution of the percentage of the 5'UTR covered at a depth of at least 30X.

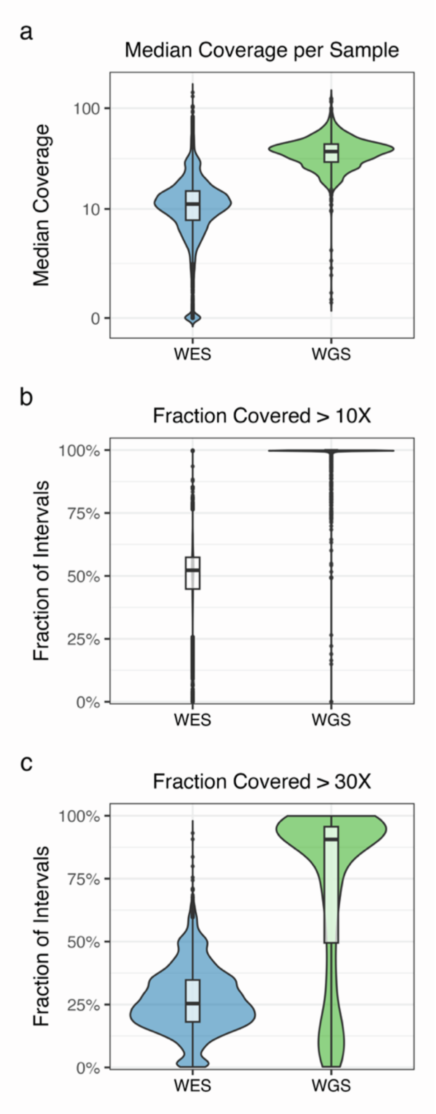

Figure S2: **Scalability analysis of 5ULTRA runtime components.** iMac runtimes were measured on an iMac18.3 equipped with a 4.2 GHz Quad-Core Intel Core i7 processor (4 cores) and 40 GB of RAM. HPC runtimes were measured on a core of an HPC computer node with 25 GB of allocatable memory running Enterprise Linux 9.

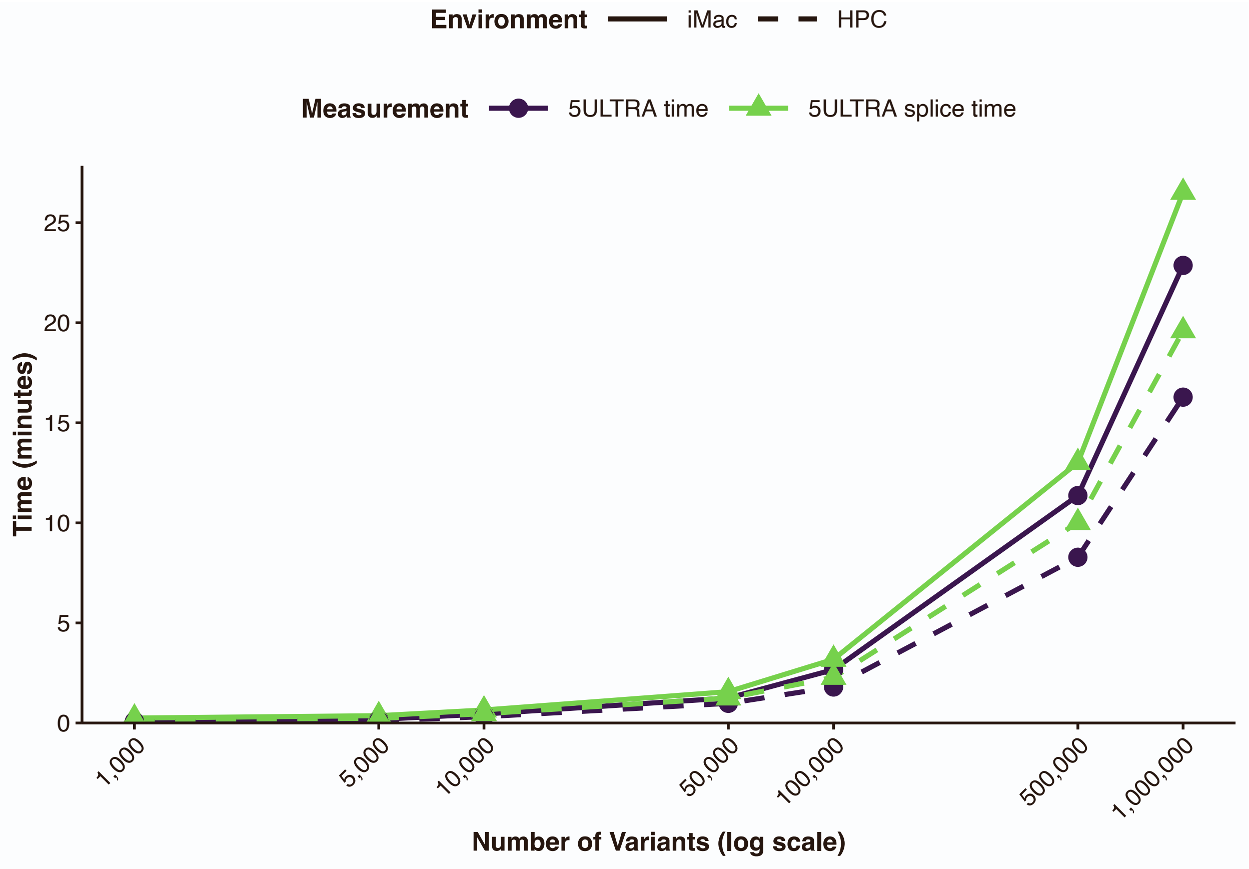

Figure S3: **Allele frequency and conservation by 5'UTR consequence.** (a) MAF distributions across variant categories. (b) PhastCons distributions. (c) PhyloP distributions.

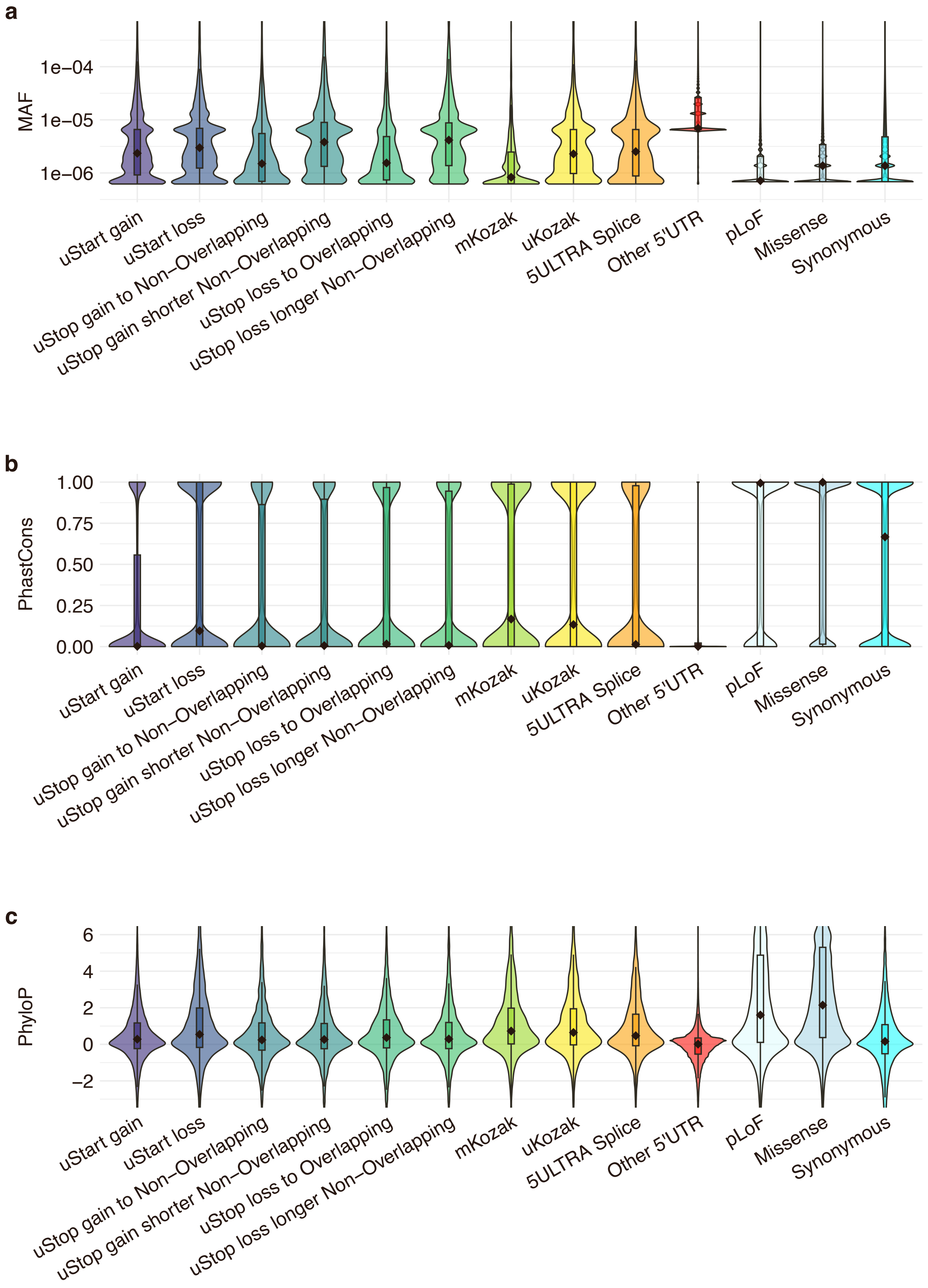

Figure S4: Feature ablation analysis. Relative importance of each feature in the predictive model. Each bar represents the decrease in the mean Area Under the Curve (AUC) from the baseline model (0.98) after the removal of that specific feature. A larger drop in AUC indicates a greater contribution of that feature to the model's overall performance. The analysis was performed using 5-fold cross-validation.

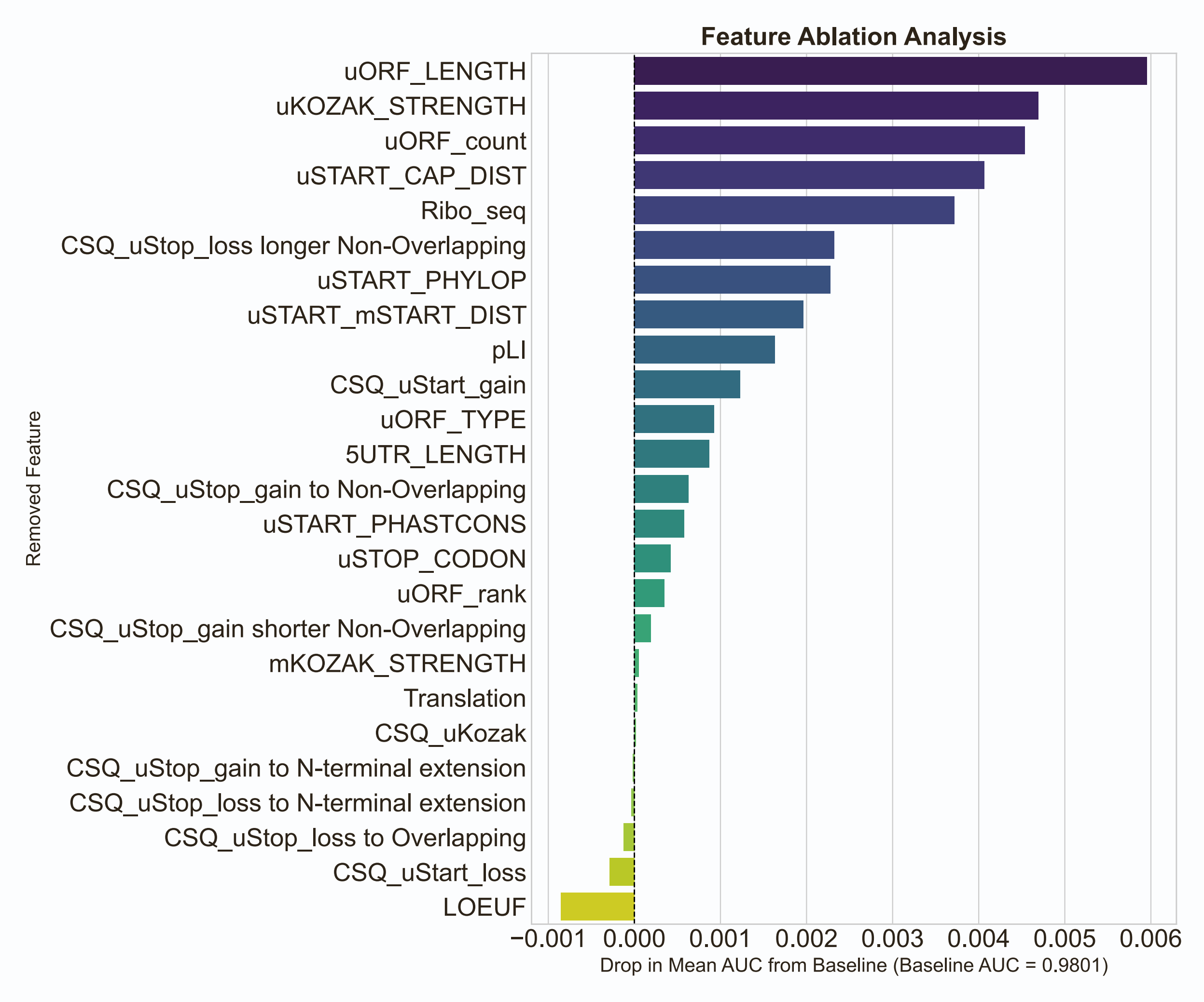

Figure S5: Comprehensive performance evaluation of 5ULTRA and CADD v1.7 on the ClinVar 5'UTR variant dataset. (a) Comprehensive performance metrics at common thresholds. This bar chart compares sensitivity, specificity, accuracy, and Matthews Correlation Coefficient (MCC). (b) Comprehensive performance metrics at common thresholds in the rare variants subset. (c) Precision-Recall (PR) curve for the pathogenic class. The plot shows precision (positive predictive value) versus recall (sensitivity) for 5ULTRA (gold) and CADD (blue). (d) Precision-Recall (PR) curve for the benign class. (e) Decision Curve Analysis (DCA). The plot displays the net benefit of using each model for clinical decision-making across a range of threshold probabilities. (f) Reliability curve comparison. This plot assesses model calibration by binning variants by deciles and plotting it against the observed proportion of pathogenic variants. The dashed line represents perfect calibration. CADD scores were min-max scaled to 0-1.

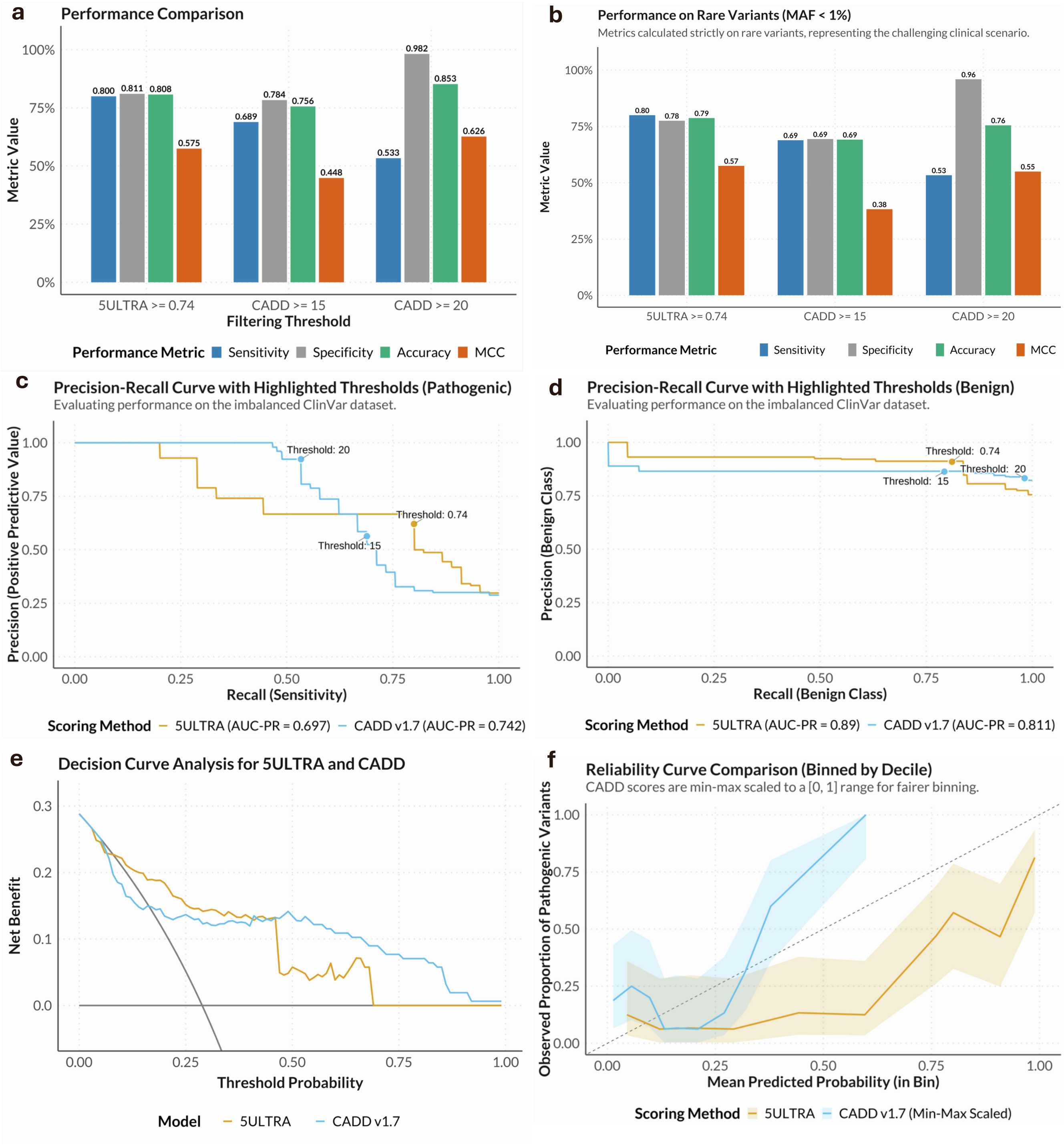
a
Performance Comparison
Metric Value
100%
75%
50%
25%
0%
0.800
0.811
0.808
0.575
0.689
0.784
0.756
0.448
0.533
0.982
0.853
0.626
5ULTRA >= 0.74
CADD >= 15
CADD >= 20
Filtering Threshold
Performance Metric Sensitivity Specificity Accuracy MCC
b
Performance on Rare Variants (MAF < 1%)
Metrics calculated strictly on rare variants, representing the challenging clinical scenario.
Metric Value
0.80
0.78
0.79
0.57
0.69
0.69
0.69
0.38
0.53
0.96
0.76
0.55
5ULTRA >= 0.74
CADD >= 15
CADD >= 20
Performance Metric Sensitivity Specificity Accuracy MCC
c
Precision-Recall Curve with Highlighted Thresholds (Pathogenic)
Evaluating performance on the imbalanced ClinVar dataset.
Precision (Positive Predictive Value)
Threshold: 20
Threshold: 0.74
Threshold: 15
Recall (Sensitivity)
Scoring Method 5ULTRA (AUC-PR = 0.697) CADD v1.7 (AUC-PR = 0.742)
d
Precision-Recall Curve with Highlighted Thresholds (Benign)
Evaluating performance on the imbalanced ClinVar dataset.
Precision (Benign Class)
Threshold: 0.74
Threshold: 20
Threshold: 15
Recall (Benign Class)
Scoring Method 5ULTRA (AUC-PR = 0.89) CADD v1.7 (AUC-PR = 0.811)
e
Decision Curve Analysis for 5ULTRA and CADD
Net Benefit
Threshold Probability
Model 5ULTRA CADD v1.7
f
Reliability Curve Comparison (Binned by Decile)
CADD scores are min-max scaled to a [0, 1] range for fairer binning.
Observed Proportion of Pathogenic Variants
Mean Predicted Probability (in Bin)
Scoring Method 5ULTRA CADD v1.7 (Min-Max Scaled)

Figure S6: 5ULTRA and CADD scores correlations with experimental translation effects from MPRA. (a) 5ULTRA vs. Delta MRL: The direction-corrected 5ULTRA score for 38 variants shows a significant and high positive correlation with the measured change in ribosome loading (Delta MRL). The plot shows the linear regression fit (blue line) with its 95% confidence interval. (b) CADD PHRED scores vs. absolute Delta MRL: a total of 1,534 5'UTR variants with a CADD PHRED score show no significant correlation with the measured absolute change in ribosome loading (absolute Delta MRL). The plot shows the linear regression fit (red line) with its 95% confidence interval.

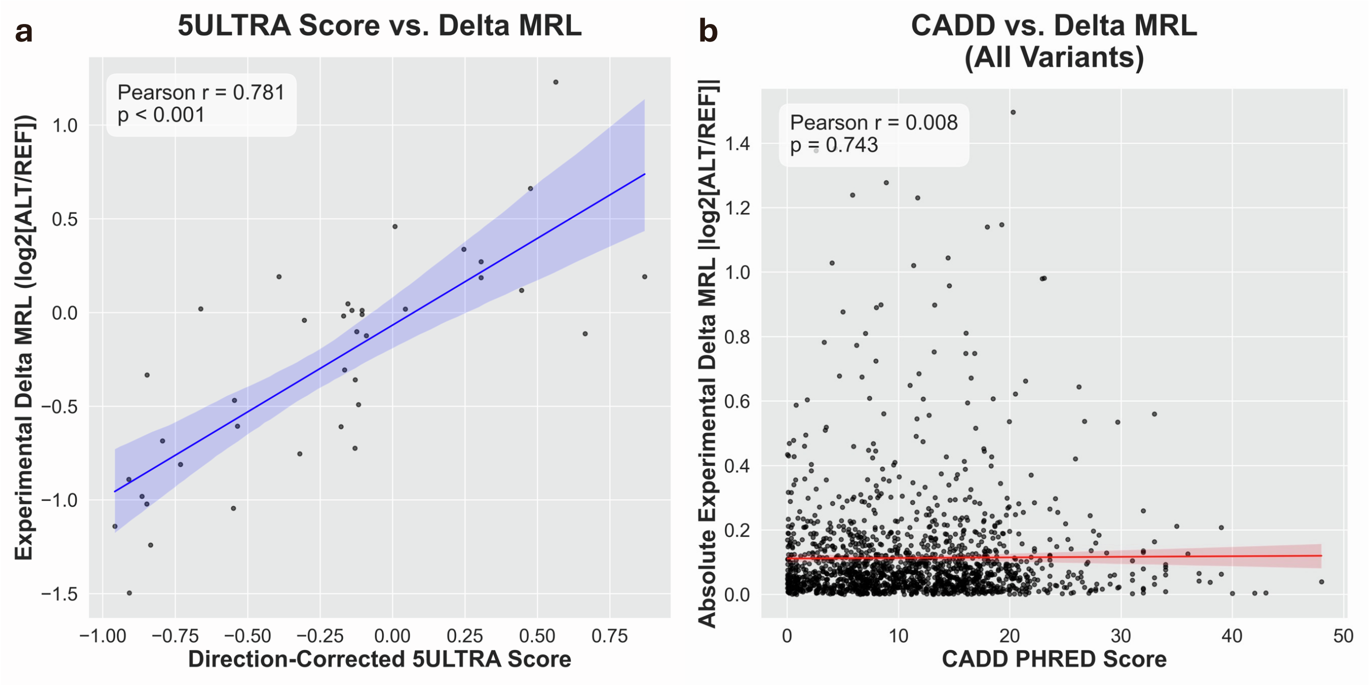

Figure S7: Enrichment in variants with high 5ULTRA scores among CGC Genes. Comparison of proportions of variants located within Cancer Gene Census (CGC) genes.

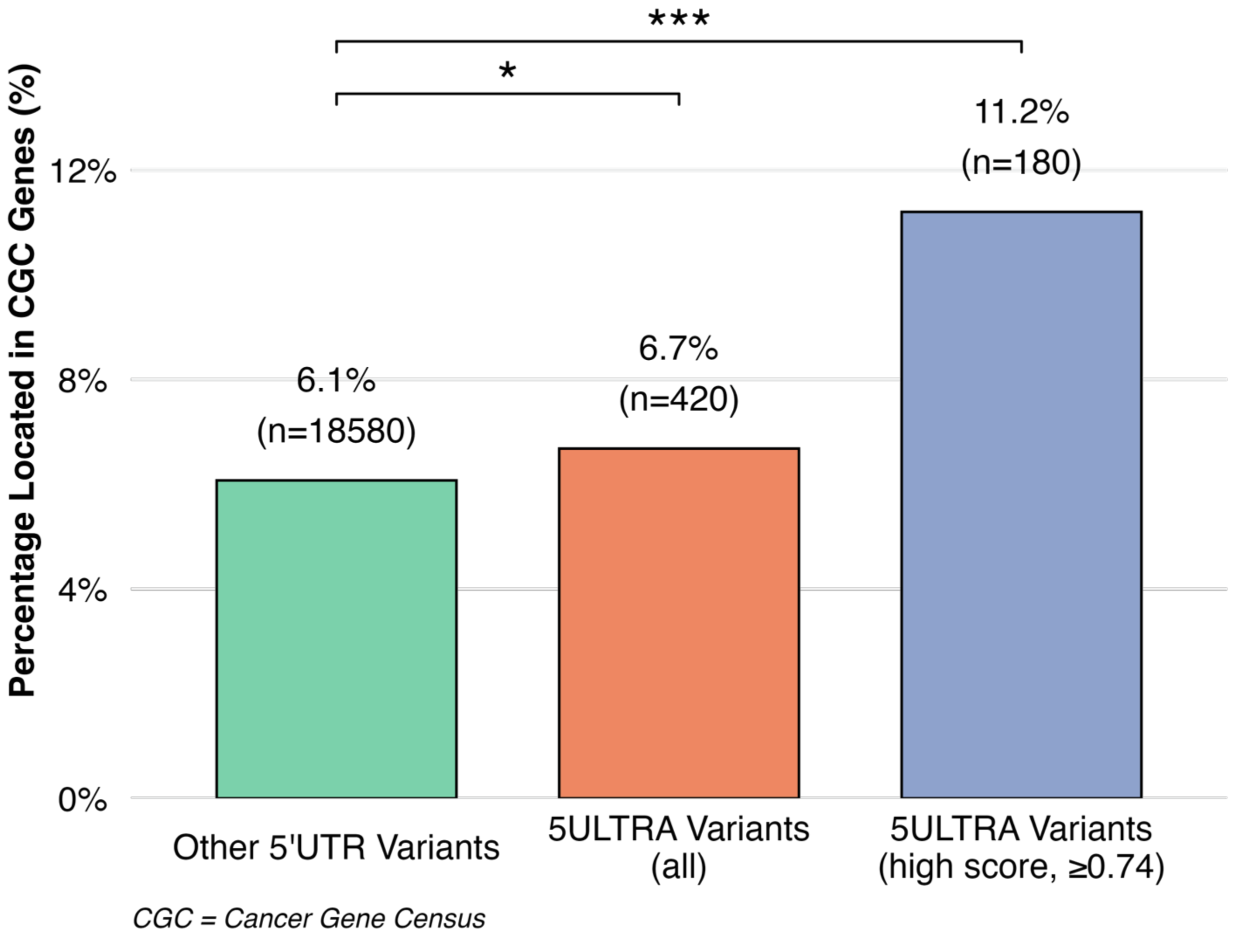

Supplemental Table Legends

Table S1: Accessory genes were defined as the union of 382 protein-coding olfactory receptor (OR) genes (Gene Ontology Molecular Function term = olfactory receptor activity [GO: 0004984]), and 190 dispensable genes.

Table S2: Features used to train the 5ULTRA score random forest model.

Table S3: 36 variants from ClinVar test set detected by 5ULTRA but missed by UTRAnnotator.

Table S4: 420 somatic variants from the COSMIC database, located in GCG genes and detected by 5ULTRA.

Table S5: 25 variants significantly associated with various phenotypes from the GWAS catalog, detected by 5ULTRA.

Supplemental Acknowledgements

H.M. was supported by the Eunice Kennedy Shriver National Institute of Child Health & Human Development of the National Institutes of Health under award number F30HD116571, and a NIGMS/NIH Medical Scientist Training Program grant T32GM152349 to the Weill Cornell/Rockefeller/Sloan Kettering Tri-Institutional MD-PhD Program. J.B. was supported by fellowships from the European Molecular Biology Organization and Marie Skłodowska-Curie Actions postdoctoral fellowship. P.D.S. and D.N.C. acknowledge financial support from Qiagen Inc through a License Agreement with Cardiff University.
