# Supplementary material for: Genome-wide detection of human 5′ UTR variants that impact protein translation
Source: Am J Hum Genet. 2026 Mar 24;113(4):809–27. doi: 10.1016/j.ajhg.2026.02.020 (PMC13087467; doi:10.1016/j.ajhg.2026.02.020)
Supplement: Document S2. Article plus supplemental information [file mmc3.pdf]

Genome-wide detection of human 5′ UTR variants that impact protein translation

Authors

Matthieu Chaldebas, Khoren Ponsin, Jonathan Bohlen, ..., Jean-Laurent Casanova, Aurélie Cobat, Peng Zhang

Correspondence

aurelie.cobat@inserm.fr (A.C.), pzhang@rockefeller.edu (P.Z.)

Chaldebas et al. develop 5ULTRA, a computational method for prioritizing 5′ UTR variants that could alter protein translation. By integrating 17 features of 5′ UTR biology into a machine-learning model, 5ULTRA identifies and prioritizes pathogenic germline and somatic 5′ UTR variants in rare and common diseases or traits.

Chaldebas et al., 2026, The American Journal of Human Genetics *113*, 809–827
April 2, 2026 © 2026 The Authors. Published by Elsevier Inc. on behalf of American Society of Human Genetics.
https://doi.org/10.1016/j.ajhg.2026.02.020

ARTICLE

Genome-wide detection of human 5′ UTR variants that impact protein translation

Matthieu Chaldebas,[1,2,3] Khoren Ponsin,[1] Jonathan Bohlen,[2,3,4,5,6] Clement Conil,[2,3] Haralambos Mourelatos,[1,7] Peter D. Stenson,[8] David N. Cooper,[8] Laurent Abel,[1,2,3] Jean-Laurent Casanova,[1,2,3,9,10,11] Aurélie Cobat,[1,2,3,11,*] and Peng Zhang[1,2,3,11,*]

Summary

The 5′ untranslated region (5′ UTR) of messenger RNAs (mRNAs) plays a central role in regulating protein synthesis initiation, particularly through the Kozak sequence and upstream open reading frames (uORFs). Genetic variants within these regulatory elements could affect translation, altering gene expression and contributing to clinical phenotypes in humans. We developed a computational method called 5ULTRA (5′ Untranslated Region Annotation) for analysis of whole-exome sequencing and whole-genome sequencing data to detect, annotate, and prioritize 5′ UTR variants with potential translation impact. 5ULTRA identifies single-nucleotide variants, indels, and splicing variants that affect uORFs by creating or disrupting start/stop codons and that alter Kozak sequence strength of either the uORFs or the main coding sequence. 5ULTRA incorporates recent uORF databases and provides comprehensive annotations. 5ULTRA implements a machine-learning score to prioritize candidate variants with predicted effects on translation and also provides specific mechanistic predictions. The score correlates strongly with experimentally measured protein-level effects of 5′ UTR variants. We applied 5ULTRA to multiple genetics datasets across diverse disease contexts, identifying candidate variants including potential cancer-driving somatic mutations predicted to decrease ABI1 level or increase NRAS abundance; common variants associated with traits such as multiple sclerosis, lung function, and cardiovascular function, by altering protein levels of TAGAP, VRTN, and SPAAR, respectively; and rare germline variants in our cohort, including a splicing variant of *RPSA* leading to 5′ UTR sequence alteration that causes congenital asplenia and a variant of *TNF* that could predispose to tuberculosis.

Introduction

The initiation of translation, by ribosome scanning of the messenger RNA (mRNA), is a crucial step in protein synthesis regulated by *cis*-acting elements encoded within the mRNA sequence. Once the ribosome is loaded onto the mRNA, it scans the 5′ untranslated region (5′ UTR) for a translation initiation site, typically an ATG codon embedded within a strong or moderate Kozak sequence context.[1,2] The Kozak motif, a key regulatory element, is defined in mammals by the consensus sequence 5′-(A/G)CCAUGG-3′.[3,4] Variants of the Kozak motif can have a major or moderate impact on ribosome translation initiation efficiency.[5] In addition, 5′ UTRs may contain upstream open reading frames (uORFs) that regulate the efficiency of translation initiation.[6–8] An uORF begins with a start codon upstream from the main coding sequence (CDS) and ends with an in-frame stop codon. After translating the uORF, the ribosome may either continue scanning in the direction of the downstream CDS (reinitiation) or may disassemble (ribosome recycling), thereby decreasing protein translation rates.[8] Ribosome stalling within uORFs can also decrease the translation efficiency of the downstream CDS.[8,9] Depending on the position of their stop codons relative to the CDS, upstream ATGs can initiate non-overlapping uORFs (terminating before the CDS), overlapping uORFs (terminating within the CDS, in a different frame), or N-terminal extensions (Figure 1A). Overlapping uORFs often have a strong inhibitory effect on mRNA translation as the ribosome traverses the start codon of the CDS, prohibiting reinitiation.[10] Some upstream ATGs produce N-terminal extensions when they are in-frame with the CDS but have no stop codon ahead of the CDS, resulting in protein products with longer sequences at their N termini, with potential impacts on protein folding, structure, and function.

The functional interpretation of non-coding variants,[11,12] including those in 5′ UTRs, is challenging due to the complex and dynamic regulatory roles of these non-coding regions.[13] Variants within the 5′ UTR, particularly those affecting Kozak motifs or uORFs, can significantly alter translation efficiency. For example, nucleotide changes at key positions of the Kozak motif (−3:R and +4:G) can influence the recognition of the start

[1]St. Giles Laboratory of Human Genetics of Infectious Diseases, Rockefeller Branch, The Rockefeller University, New York, NY 10065, USA; [2]Paris Cité University, Imagine Institute, 75015 Paris, France; [3]Laboratory of Human Genetics of Infectious Diseases, Necker Branch, INSERM UMR1163, 75015 Paris, France; [4]Gene Center and Department of Biochemistry, Ludwig-Maximilians-Universität, Munich, Germany; [5]Department of Pediatrics, Dr. von Hauner Children's Hospital, University Hospital, Ludwig-Maximilians-University Munich, Munich, Germany; [6]German Center for Child and Adolescent Health (DZKJ), Munich, Germany; [7]Weill Cornell/Rockefeller/Memorial Sloan Kettering Tri-Institutional MD-PhD Program, New York, NY 10021, USA; [8]Institute of Medical Genetics, School of Medicine, Cardiff University, Cardiff CF14 4XN, UK; [9]Department of Pediatrics, Necker Hospital for Sick Children, Paris, France; [10]Howard Hughes Medical Institute, New York, NY 10065, USA
[11]These authors contributed equally
*Correspondence: aurelie.cobat@inserm.fr (A.C.), pzhang@rockefeller.edu (P.Z.)
https://doi.org/10.1016/j.ajhg.2026.02.020.
© 2026 The Authors. Published by Elsevier Inc. on behalf of American Society of Human Genetics.
This is an open access article under the CC BY license (http://creativecommons.org/licenses/by/4.0/).

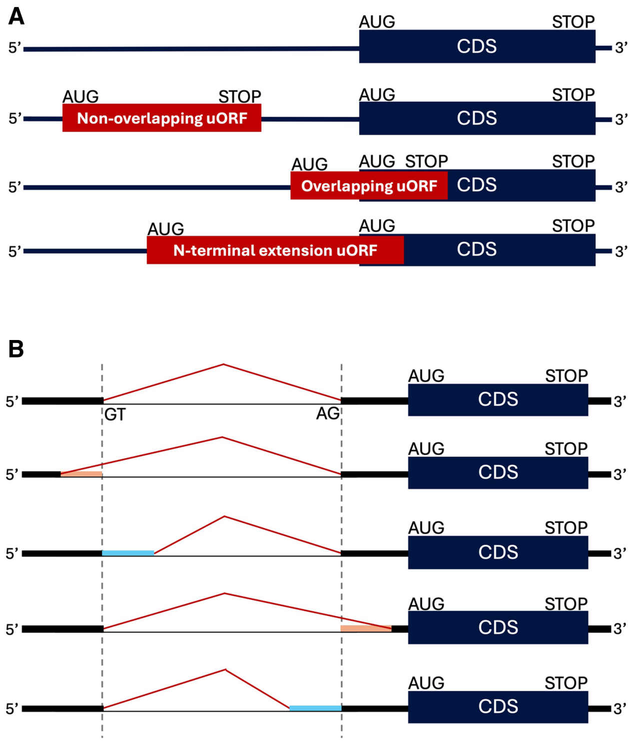

Figure 1. uORF types and splicing alterations in 5′UTR
(A) uORF types: non-overlapping (entirely upstream from the CDS), overlapping (partially overlapping the CDS), and N-terminal extension (beginning upstream and extending into CDS).
(B) 5′ UTR splicing variant effects: wild type; alternative 5′ splice site: deletion (orange) or intron retention (blue); and alternative 3′ splice site: deletion (orange) or intron retention (blue).

codon by ribosomes.[5] The first pathogenic Kozak variant was reported in 1985 as the cause of alpha-thalassemia in three individuals.[14] Variants that create, eliminate, or modify uORFs can also affect mRNA translation. A newly created upstream start codon may introduce a uORF that decreases the rate of mRNA translation by triggering ribosome disassembly or by diverting ribosomes away from the CDS, which was first reported as a pathological mechanism in individuals with beta-thalassemia in 1991.[15] Conversely, the removal of an uORF can enhance translation by allowing more ribosomes to reach the CDS. Variants that convert a non-overlapping uORF into an overlapping uORF reduce translation rates further by preventing reinitiation. There are several factors that could affect translation efficiency, such as the distance from the 5′ end of the mRNA to the start of the uORF, the strength of the Kozak sequence around the start codons of the uORF and the CDS, conservation of the uORF, and the presence of multiple uORFs.[10,16] The type of uORF stop codon (TAA, TAG, or TGA) can also affect translation to some extent by affecting the release of ribosomes from the mRNA.[17–19] These studies have highlighted the intricate and complicated regulatory function of 5′ UTRs and the potential impact of 5′ UTR variants on gene expression and protein synthesis. Variants in 5′ UTRs, particularly in dosage-sensitive genes, could therefore have functional impact, underlying pathological conditions.[20–23]

However, the interpretation of these variants is challenging, and they are often overlooked in conventional analyses. Recent studies have suggested that a careful review of their classification in public databases is required.[24] Computational approaches for addressing this have emerged. Tools such as MORFEE,[25,26] UTRAnnotator,[27] and utr.annotation[28] focus on annotating variants based on their effects on known regulatory elements, such as uORFs. However, these useful tools are subject to several limitations. First, they are based on small databases of uORFs,[29] while larger and more comprehensive databases have since emerged, including the uORFdb database, which includes computationally identified uORFs,[30] and the Ribo-uORF database, which focuses on uORFs confirmed by ribosome sequencing (ribo-seq).[31] Second, the available tools support single-nucleotide variants (SNVs) and small indels (1–5 nucleotides) but exclude larger variants with a potential impact on 5′ UTRs. Third, some types of 5′ UTR variants affecting translation, such as those affecting splicing within 5′ UTRs, are not supported by these tools. Fourth, these tools lack annotations and scores that are crucial for variant interpretation and prioritization, as some specific features have been shown with effects on translation regulations (e.g., the uORF stop codon, evolutionary conservation of the uORF start codon, and the length of the 5′ UTR).[8,16,17,19,22,32] In this context, we aimed to develop a computational approach for detecting a broader range of 5′ UTR variants, with enriched annotations and a scoring function to predict their impact on protein translation dynamics. This tool will improve our ability to identify candidate 5′ UTR variants, providing insight into the genetic etiology of human diseases.

Material and methods

5′ UTR and uORF data

We obtained human genome sequence and gene annotation data for the GRCh38/hg38 genome assembly from the GENCODE v45 database,[33] focusing on 5′ UTRs in genes/transcripts tagged as "basic." We collected data from the recently published Ribo-uORF database, integrating 1,495 curated ribo-seq datasets containing 501,554 actively translated uORFs. We also retrieved uORF data from the uORFdb and sorfs.org databases. In this study, we focused on uORFs beginning with the canonical ATG codon. After mapping the uORFs onto the 5′ UTRs, we retained 79,394 uORFs from 62,312 5′ UTRs. All these data for uORFs and 5′ UTRs and their features were integrated into the output of our computational method 5ULTRA (5′ Untranslated Region Annotation). Our analysis focused on 18,775 MANE (Matched Annotation from NCBI and EMBL-EBI) transcripts from 18,749 protein-coding genes. This curated set represents a single, well-supported transcript for each protein-coding gene that is perfectly matched between Ensembl/GENCODE and NCBI RefSeq. We chose the MANE set as our reference for several key reasons: standardization, biological relevance, and reproducibility.

Gene group definition for comparative analysis

We categorized three groups of genes for the comparative analysis of variants detected by 5ULTRA: Human Gene Mutation Database (HGMD) genes were defined as the 6,817 human genes known to be linked to disease, with at least one disease-causing mutation (DM) listed in the HGMD database (regardless of the location of that mutation within the gene); Accessory genes were defined as the union of 382 protein-coding olfactory receptor (OR) genes (Gene Ontology molecular function term = olfactory receptor activity [GO:0004984]) and 190 dispensable genes[34] (Table S1) absent from HGMD; the remaining 11,632 genes were categorized as "Other genes."

Significance

Significance levels for pairwise differences are indicated in the figures by asterisks, as follows: *$p < 0.05$, **$p < 0.01$, and ***$p < 0.001$.

Pathway enrichment analysis

Gene set enrichment analysis was performed with Reactome pathways (v.2024.1). Over-representation was assessed in Fisher's exact tests, and p values were corrected by the Benjamini-Hochberg procedure.

5ULTRA development and annotation

We developed 5ULTRA in Python (v.3.13.1) and made it available from https://github.com/casanova-lab/5ULTRA. For each variant, 5ULTRA checks whether the variant falls within the boundaries of at least one 5′ UTR, evaluates six categories of consequences (uStart gain, uStart loss, uStop gain, uStop loss, uKozak, and mKozak), and annotates the variants. For Kozak variants, only the −3 and +4 positions are considered. For the detection of potential perturbations of splice-site selection, we used SpliceAI v.1.3[35] to generate precomputed scores for all possible SNVs within 100 bp upstream or downstream of each canonical splice site within the 5′ UTR. We included an "all transcripts" option in the 5ULTRA tool. This feature allows for a more comprehensive and exploratory analysis of variants that fall outside the canonical MANE set but inside a protein-coding transcript.

Human population variants and minor allele frequencies

We retrieved variant data from gnomAD v.4.1.0, encompassing 76,215 whole-genome sequencing (WGS) and 734,947 whole-exome sequencing (WES) sequences.[36] Minor allele frequency (MAF) values were obtained from the gnomAD v.4.1 joint dataset. MAF values for the coding-region variants used for comparison (predicted loss of function [pLoF], missense, and synonymous) were obtained from the gnomAD v.4.1 exomes dataset. Initial processing involved filtering the variants to retain only those located within MANE transcripts, with non-zero allele frequency information and FILTER labeled as PASS. Finally, we ensured that the categories were distinct by identifying variants found simultaneously in the 5ULTRA, 5ULTRA splice, other 5′ UTR, missense, pLoF, and synonymous datasets and removing them from these specific sets. The final set comprised 133,823 5ULTRA variants, 3,754 5ULTRA splice variants, 27,430,206 other 5′ UTR variants, 1,399,456 pLoF variants, 10,925,786 missense variants, and 5,072,749 synonymous variants. MAF distributions were compared in two-tailed Wilcoxon rank-sum tests. The Hodges-Lehmann estimator was used to quantify the median difference in MAF for variants between the HGMD and accessory gene sets.

Cross-species conservation scores

We obtained the phyloP-100way and PhastCons-100way genome-wide cross-species conservation scores from the UCSC Genome Browser[37] based on the alignments of sequences from 100 vertebrates. PhyloP measures conservation at individual alignment positions, independently of neighboring sites. This approach is useful for detecting signatures of selection at specific nucleotides or classes of nucleotides. PhyloP scores represent $-\log p$ values under a null hypothesis of neutral evolution, with positive scores indicating conservation (evolution slower than expected) and negative scores indicating acceleration (faster evolution than expected).[38] PhastCons employs a hidden Markov model to estimate the probability that each nucleotide belongs to a conserved element.[38] This method considers both the individual alignment positions and the flanking positions and is therefore effective for identifying conserved regions. PhastCons scores range from 0 to 1, representing the probability of negative selection.

5ULTRA score training and test datasets

We trained and evaluated a random forest model to predict the functional impact of 5′ UTR variants involving uORFs. We screened HGMD Professional v.2025.1[39] and extracted 1,695 5′ UTR variants labeled as "DM" (i.e., disease-causing mutations), 202 of which affected uORFs or Kozak sequences. After excluding 27 splicing variants and 12 variants affecting only the main Kozak sequence (mKozak), the remaining 163 variants affecting uORFs were used as positive controls to train the model. Similarly, 440 common variants from gnomAD v.4.1 (allele frequency >0.05) were used as negative controls to train the model. We tested the model on an independent dataset, from ClinVar 2024-05-02.[40] A total of 6,118 5′ UTR variants, classified as "pathogenic" or "benign," were retained for testing after removal of the variants already present in the training set. 5ULTRA scores were compared to CADD v.1.7, and to UTRAnnotator that was used through the plug-in of VEP v.109.3.

Feature engineering and processing

Datasets were preprocessed to include 5′ UTR variants located within MANE transcripts, excluding variants with "mKozak" annotations. Overlapping variants were removed sequentially: HGMD overlaps were removed from ClinVar and gnomAD, then ClinVar overlaps were removed from gnomAD, based on genomic coordinates. The features considered are summarized in Table S2 and included 5′ UTR context (5′ UTR length, uAUG-AUG distance, 5′ end-uAUG distance, and uORF rank/length), uORF characteristics (type, uStop codon, and uKozak strength), evolutionary conservation (PhyloP and PhastCons at uAUG), gene constraint (pLI and LOEUF), ribosome profiling evidence of translation, mKozak strength, and variant consequence (CSQ). Continuous missing values were imputed with the median value from the training set. Categorical features were label encoded, and CSQ was one-hot encoded.

Model training and hyperparameters

We used SMOTE before the training for class balancing, and hyperparameters were tuned using 5-fold cross-validation and fixed as follows: n_estimators = 500, class_weight = "balanced," random_state = 42, max_depth = none, max_features = none, min_samples_leaf = 2, min_samples_split = 2. Model performance was assessed by 5-fold stratified cross-validation. The probability of an effect on mRNA translation predicted by this model was defined

as the 5ULTRA score, ranging from 0 (no effect on translation of the CDS) to 1 (strong effect on translation of the CDS).

pQTL analysis

Genetic association data for circulating protein levels were obtained from publicly available summary statistics generated by Hawkes et al.[41] These statistics originate from a large-scale WGS study on approximately 50,000 UK Biobank participants.[42] The original study involved *cis*-association analyses between 1.1 billion genetic variants and 2,907 circulating protein levels, using both single-variant and aggregate-based testing approaches. For our analyses, we used all the variants present in the summary statistics from the discovery cohort, which were derived from 46,362 individuals of inferred European genetic ancestry. We applied a Benjamini-Hochberg false discovery rate (FDR) correction (adjusted p value <0.05) to the summary statistics. We assessed the correlation of 5ULTRA score with *cis*-protein quantitative trait locus (*cis*-pQTL) effect size by assigning a positive sign to the 5ULTRA score for predictions of "increases" in mRNA translation and a negative sign for predictions of "decreases" in translation.

MPRA variant data

Delta mean ribosome load (ΔMRL) was calculated for SNVs using the GSE114002 dataset,[43] sample GSM3130443, by computing the $\log_2$ fold change of the variant MRL relative to the reference MRL ($\log_2[MRL_{alt}/MRL_{ref}]$). To evaluate the relationship between experimental translational effects and 5ULTRA, ΔMRL values were correlated with 5ULTRA scores. 5ULTRA scores were direction corrected (negated for variants predicted to decrease translation) to align with ΔMRL directionality. Statistical associations were quantified using Pearson correlation coefficients (r) and visualized via linear regression.

Somatic variant data

We collected more than 300,000 5′ UTR variants (labeled "confirmed somatic variant") documented in the COSMIC v.99 database, an expert-curated knowledge base containing data for somatic variants in cancer.[44] These somatic variants were detected in human cancer tissues from various sources (>29,000 scientific publications and large studies). We focused on 748 genes listed in the COSMIC Cancer Gene Census.[45]

GWAS variant data

We obtained the data from NHGRI-EBI GWAS catalog v.1.0.3.1, a curated, structured and standardized database providing summarized information for >45,000 published genome-wide association studies (GWASs) and across >5,000 human traits.[46] We extracted a list of 533,484 genetic loci associated with common traits and diseases identified in different GWASs.

Data visualization

Data were manipulated and visualized with the data.table, dplyr, ggpubr, ggsignif, tidyverse, and ggplot2 packages in R 4.4.0.

Results

Genome-wide analysis of uORF sequences and Kozak motifs in 5′ UTRs

We investigated the prevalence and functional roles of uORFs in human genes by performing a genome-wide analysis of uORF sequences and Kozak sequence motifs. We integrated 5′ UTR data from GENCODE v.45[33] for 18,775 MANE protein-coding transcripts[47] with the uORF databases.[29–31] We identified 22,567 uORFs located within these 5′ UTRs, 8,067 (35.7%) of which were supported by ribo-seq evidence of translation. The median 5′ UTR length was 136 nt (Figure 2A), and 42.5% contained at least one uORF (Figure 2B). We hypothesized that uORF distribution might vary with gene function. In support of this hypothesis, 6,817 genes with known disease-causing variants (from HGMD Professional v.2025.1[39]) contained significantly fewer uORFs than 513 accessory genes (protein-coding olfactory receptors and dispensable genes,[34] see material and methods) ($p < 2 \times 10^{-16}$, Figure 2C), suggesting that disease-relevant genes are subject to stronger selection pressure to maintain translational efficiency. This difference remained significant ($p = 6.99 \times 10^{-9}$) in the analysis comparing genes with known disease-causing variants and all the remaining 11,632 genes. Pathway enrichment analysis (Figure 2D) provided additional evidence of functional differences. Indeed, the olfactory signaling pathway was significantly enriched in uORF-containing genes ($p = 1.29 \times 10^{-37}$), probably due to more relaxed selective constraints. Conversely, pathways essential for rapid immune responses, such as neutrophil degranulation ($p = 4.9 \times 10^{-24}$), were significantly depleted of uORF-containing genes, suggesting a requirement for highly efficient translation that renders the presence of uORFs less likely. An analysis of Kozak motifs revealed a significant difference ($p < 2.2 \times 10^{-16}$, Figure 2E) of the star codons between uORFs and CDS. A higher proportion of uORFs had weak Kozak motifs, suggesting that uORFs have a less efficient translation initiation than CDS.

5ULTRA: 5′ UTR variants that could affect protein translation

We addressed the need for an improved annotation and prioritization of variants within 5′ UTRs that could affect translation by developing 5ULTRA, a computational method for identifying and characterizing such variants in protein-coding genes. 5ULTRA aims to identify variants with a high likelihood of affecting protein translation through comprehensive qualitative annotations and a quantitative score. 5ULTRA accepts variants in VCF format and evaluates their impact on six key categories of consequences: (1) uORF creation (uStart-gain); (2) existing uORF start codon disruption (uStart-loss); (3) premature uORF termination (uStop-gain); (4) uORF stop codon disruption (uStop-loss); (5) alteration of the strength of existing uORF Kozak sequences (uKozak); and (6) changes in CDS Kozak sequence strength (mKozak). 5ULTRA annotates both SNVs and indels, providing details of uORF sequence, length, position, conservation, and transcript features (Box 1). These detailed annotations are integrated into a machine-learning-derived score that quantifies the predicted functional impact of each variant to facilitate the prioritization of candidate variants (see material and methods). 5ULTRA is designed for seamless integration

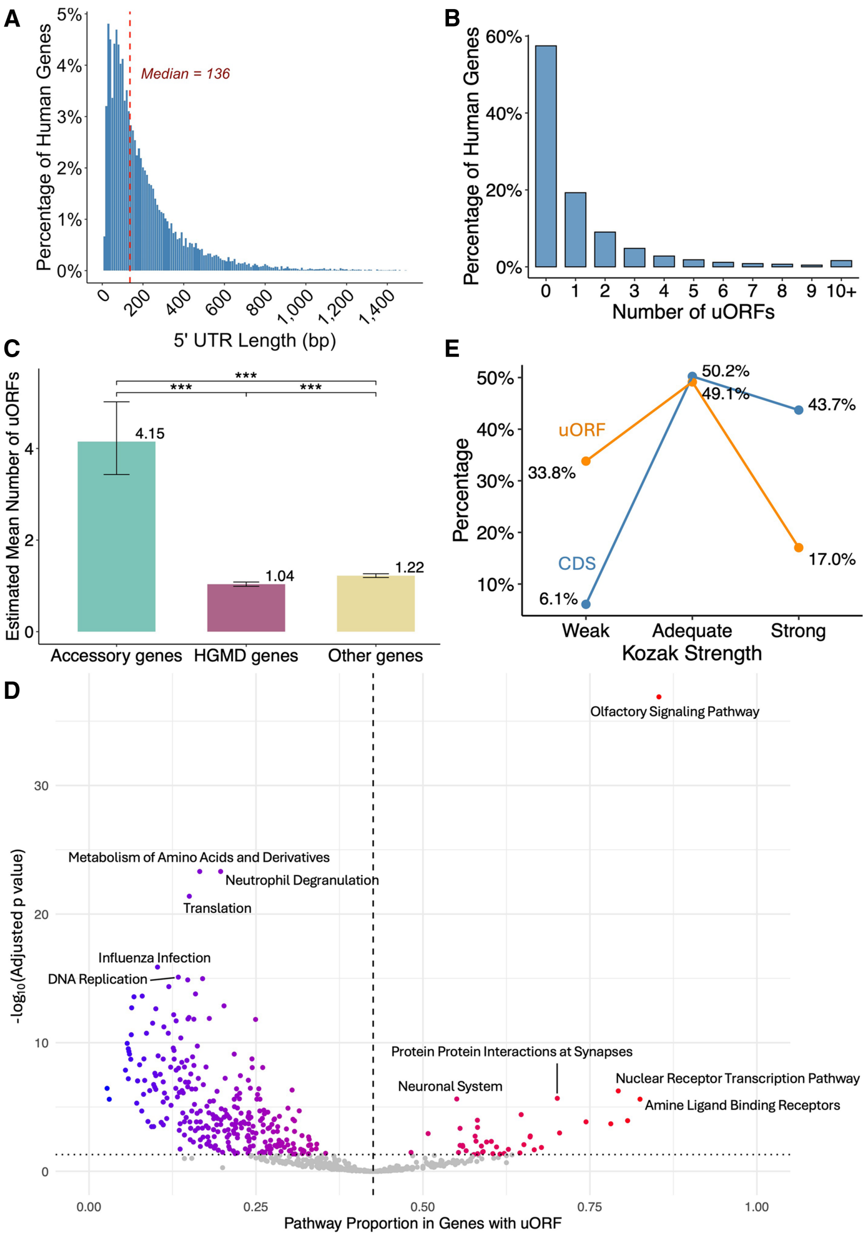

Figure 2. Characterization of 5′ UTR sequences

Analysis of 18,775 MANE protein-coding transcripts.

(A) 5′ UTR length distribution (nucleotides), with median.

(B) uORF count proportions.

(C) Mean number of uORFs per gene for accessory, HGMD, and other genes (see material and methods; 95% confidence intervals, negative binomial regression, Tukey-adjusted pairwise comparisons).

(D) Pathway enrichment for genes with uORFs (see material and methods).

(E) Kozak motif strength comparison (uORF vs. CDS start codons; chi-squared test, $p < 2.2 \times 10^{-16}$).

into WES and WGS pipelines via a single-command-line approach. Worth to note that WGS typically provides more comprehensive and uniform coverage of 5′ UTR intervals than WES (Figures S1A–S1C). Computational performance scales efficiently and linearly with the number of input variants, making it possible to analyze large datasets (Figure S2). 5ULTRA is also accessible via a user-friendly webserver interface. The comprehensive 5′ UTR and uORF datasets used in this study are publicly available for download, serving as a resource for the research community.

Exonic and intronic variants may alter splicing and thus modify 5′ UTR sequences

Alteration of 5′ UTR sequences arise not only from direct variants within the 5′ UTR itself but also from missplicing events. We therefore incorporated a splicing-aware analysis module into 5ULTRA. The start codon of the CDS resides within the first exon in 11,675 (62.2%) MANE transcripts, whereas in the remaining 7,100 (37.8%) transcripts, it is present in downstream exons. In these cases, the mature 5′ UTR is formed after the completion of splicing. Notably, 140 transcripts were found to have their start codons located precisely at the start of the second exon, such that the entire first exon is part of the 5′ UTR. Thus, variants in upstream exons or introns can alter 5′ UTR composition through aberrant splicing, potentially affecting translation. 5ULTRA addresses this issue by integrating the SpliceAI algorithm,[35] which screens variants for the probability to alter splicing. SpliceAI identifies variants that may create or damage 5′ or 3′ splice sites (5′ss/3′ss) within a 100-nt window around canonical splice sites, using a cutoff of ≥0.2 for high sensitivity. Based on the predicted alternative splice sites, 5ULTRA treats these variants as insertions or deletions relative to the wild-type transcript, reconstructing the altered 5′ UTR sequence (Figure 1B). This altered 5′ UTR sequence is then analyzed for changes in uORF and Kozak sequence context, using the same six consequence categories described above (uStart-gain/loss, uStop-gain/loss, uKozak, and mKozak). Two splicing-related features are added to the output to distinguish variants identified through this splicing module (Box 1). This approach enables 5ULTRA to comprehensively identify and characterize variants with potential impacts on translation, either directly within the 5′ UTR or indirectly through altered splicing.

Box 1. Input and output of 5ULTRA software

5ULTRA takes variants in VCF format as input, with its first five columns as mandatory fields (CHROM, POS, ID, REF, and ALT). It supports human reference genome GRCh38/hg38 and provides an option for all transcripts (based on the GENCODE database[33]) or canonical transcripts (based on the MANE database[47]). It outputs SNVs and indels that may affect translation, with the following annotations.

- CSQ: consequence type of variant
- Translation: effect on CDS translation (increased, decreased, N-terminal extension)
- 5ULTRA_Score: prioritization metric
- GENE: gene symbol
- TRANSCRIPT: Ensembl transcript ID (e.g., ENST123456789.1)
- MANE: NCBI transcript ID if applicable (e.g., NM_123456789.1)
- 5UTR_START: genomic position of the 5′ UTR start
- 5UTR_END: genomic position of the 5′ UTR end
- STRAND: DNA strand (+ or −)
- 5UTR_LENGTH: length of the 5′ UTR
- START_EXON: CDS start exon position
- mKOZAK: nucleotide sequence −4 to +5 around the main CDS start
- mKOZAK_STRENGTH: Kozak strength (Weak, Adequate, Strong, or NA) of the main CDS
- uORF_count: total number of uORFs in the transcript
- Overlapping_count: number of overlapping uORFs
- Nterminal_count: number of N-terminal extension uORFs
- NonOverlapping_count: number of non-overlapping uORFs
- uORF_START: genomic position of the uORF start
- uORF_END: genomic position of the uORF end
- Ribo_seq: evidence of translation (True, False, or New uORF)
- uSTART_mSTART_DIST: distance from the uORF start to the CDS start
- uSTART_CAP_DIST: distance from the uORF start to the 5′ UTR cap
- uSTOP_CODON: uORF stop codon (TAA, TGA, or TAG)
- uORF_TYPE: uORF type (Non-overlapping, Overlapping, N-terminal extension)
- uKOZAK: nucleotide sequence −4 to +5 around the uORF start
- uKOZAK_STRENGTH: Kozak strength (Weak, Adequate, Strong, or NA) of the uORF
- uORF_LENGTH: length of the uORF
- uORF_AA_LENGTH: length of the uORF in amino acids
- uORF_rank: rank of the uORF based on proximity to the CDS start
- uSTART_PHYLOP: mean conservation score of the uORF start (PhyloP)
- uSTART_PHASTCONS: mean conservation score of the uORF start (PhastCons)
- pLI: probability of the gene being intolerant to losses of function
- LOEUF: gene loss of function observed/expected upper bound fraction

Splice module additional columns:

- SpliceAI: SpliceAI predictions for the variant
- Splicing_CSQ: consequence of mis-splicing for the 5′ UTR sequence

Annotating human population 5′ UTR variants and their evolutionary conservation

We investigated the population-level frequency of 5′ UTR variants predicted to affect translation by analyzing a total of 27,567,783 variants within the 5′ UTRs of MANE transcripts, which were identified from 76,215 WGSs and 734,947 WESs in gnomAD v.4.1.0.[36] 5ULTRA predicted that 137,577 of these genetic variants would affect translation by modifying uORFs or Kozak sequences, 3,754 of which were predicted to affect splicing. The 133,823 non-splicing variants were classified as follows (Figure 3A): 52,236 uStart-gain (39.5%), 29,394 uStart-loss (22.3%), 20,849 uStop-loss (15.8%), 16,945 uStop-gain (12.8%), 6,582 uKozak (5.0%), and 6,081 mKozak (4.6%). The predicted effects of the 3,754 splicing variants were 1,543 uStart-gain (41.1%), 686 uStop-gain (18.3%), 659 uStop-loss (17.6%), 615 uStart-loss (16.4%), 229 mKozak (6.1%), and 22 uKozak (0.6%). We hypothesized that the variants with a functional impact identified by 5ULTRA would have lower allele frequencies due to stronger purifying selection. Consistent with this hypothesis, 5ULTRA variants had a significantly lower median MAF (2.34×10^{-6}) than

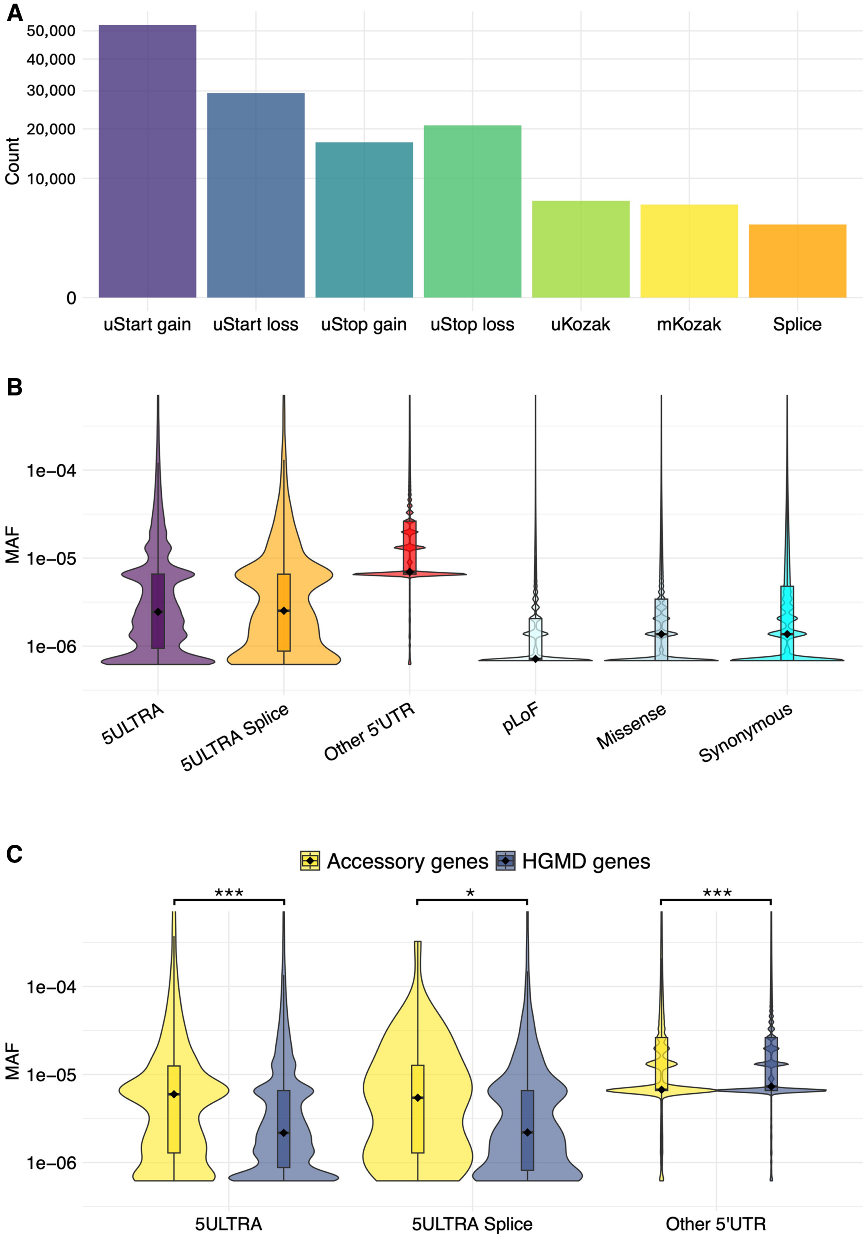

Figure 3. Analysis of the frequency of the 137,289 5′ UTR gnomAD variants
(A) Distribution of functional consequences as predicted by 5ULTRA.
(B) MAF distributions across variant categories (5ULTRA-annotated, 5ULTRA splice module, other 5′ UTR, and coding [pLoF, missense, and synonymous]).
(C) MAF comparison (5ULTRA-annotated vs. other 5′ UTR), stratified by gene group (HGMD, accessory; Mann-Whitney U test, Benjamini-Hochberg adjusted p values).

other 5′ UTR variants (6.59×10^{-6}; $p < 2.2 \times 10^{-16}$, Wilcoxon rank-sum test) (Figure 3B). Notably, the subset of 5ULTRA variants affecting the main CDS Kozak sequence (mKozak) had the lowest median MAF (8.29×10^{-7}), significantly lower than missense and synonymous coding variants (median MAF = 1.37×10^{-6}; $p < 2.2 \times 10^{-16}$) (Figure S3A). A deeper analysis showed that uStop-loss and uStop-gain variants altering uORF type (e.g., overlapping to non-overlapping) had a lower MAF than those affecting uORF length only (Figure S3A). Thus, uORF-altering variants causing more substantial changes in uORF features have a lower frequency, implying a greater functional significance. We compared MAF distributions between disease-associated genes (from HGMD) and accessory genes (protein-coding olfactory receptor and dispensable genes,[34] see material and methods; Figure 3C). The 5ULTRA variants had significantly lower MAFs in HGMD genes than in the accessory genes ($p < 2 \times 10^{-16}$, estimated median difference = -1.6×10^{-5}), suggesting that selection pressure is stronger in disease-relevant contexts. By contrast, other 5′ UTR variants displayed a lower MAF in the accessory genes ($p < 2 \times 10^{-16}$, estimated median difference = 2.1×10^{-11}), indicating a weaker dependency of their frequency on the disease association status. An analysis of conservation revealed significantly higher PhastCons and PhyloP[38] scores for 5ULTRA-annotated variants than for other 5′ UTR variants (Figures S3B and S3C), suggesting greater cross-species conservation and stronger functional constraint.

5ULTRA score for the prioritization of candidate 5′ UTR variants

We developed the 5ULTRA score, a prioritization metric (ranging from 0 to 1) for 5′ UTR variants affecting uORFs, using a machine-learning random forest classifier.[48] The model was trained on HGMD disease-causing variants[39] as positive controls vs. common variants (MAF > 5%) from gnomAD[36] as negative controls. In total, 163 of the HGMD variants specifically involved uORFs (excluding mKozak and splicing variants). Experimental biochemical evidence had been published for 42 of these variants (Table 1); 37 (88%) had been reported to have translational effects consistent with 5ULTRA predictions, and 5 (12%) affected transcription (e.g., altering promoter/transcription factor binding sites [TFBSs]) or affected the CDS of a non-canonical transcript. This high degree of agreement supports the use of HGMD variants for training. The negative training set comprised 440 common gnomAD variants (MAF > 5%) affecting uORFs (excluding mKozak and

Table 1. 42 germline 5′ UTR variants detected by 5ULTRA and associated with disease, with supporting functional evidence

CHROM	POS	REF	ALT	HGVS	Gene	Disease	CSQ	MAF	PubMed ID Year
1	42958758	C	T	c.107G>A (GenBank: NM_006516.4)	*SLC2A1*	glucose transporter type 1 deficiency syndrome	uStart_gain	0	Willemsen et al.[49] 2017
2	25164783	G	T	c.11C>A (GenBank: NM_000939.4)	*POMC*	obesity, adrenal insufficiency and red hair	uStart_gain	2.54 × E-05	Krude et al.[50] 1998
2	202376528	GC	AT	c.947−946delinsAT (GenBank: NM_001204.7)	*BMPR2*	pulmonary hypertension	uStart_gain	0	Aldred et al.[51] 2007
3	93973788	G	A	c.39C>T (GenBank: NM_000313.4)	*PROS1*	protein S deficiency	uStart_gain	6.39 × E-07	Labrouche-Colomer et al.[52] 2020
3	184376290	C	A	c.31G>T (GenBank: NM_000460.4)	*THPO*	thrombocythemia, essential	uStop_gain	0	Ghilardi et al.[53] 1999
5	14871458	G	A	c.11C>T (GenBank: NM_054027.6)	*ANKH*	chondrocalcinosis 2	uStart_gain	0	Pendleton et al.[54] 2002
5	36876801	GA	AT	c.457−456delinsAT (GenBank: NM_133433.4)	*NIPBL*	Cornelia de Lange syndrome	uStart_gain	0	Coursimault et al.[55] 2022
5	88823796	G	A	c.8C>T (GenBank: NM_002397.5)	*MEF2C*	MEF2C haploinsufficiency syndrome	uStart_gain	0	Wright et al.[56] 2021
5	88823814	G	A	c.26C>T (GenBank: NM_002397.5)	*MEF2C*	MEF2C haploinsufficiency syndrome	uStart_gain	0	Wright et al.[56] 2021
5	88823854	T	A	c.66A>T (GenBank: NM_002397.5)	*MEF2C*	MEF2C haploinsufficiency syndrome	uStart_gain	0	Wright et al.[56] 2021
5	88823891	C	T	c.103G>A (GenBank: NM_002397.5)	*MEF2C*	MEF2C haploinsufficiency syndrome	uStart_gain	0	Wright et al.[56] 2021
5	132369824	G	A	c.149G>A (GenBank: NM_003060.4)	*SLC22A5*	carnitine deficiency, primary	uStart_gain	2.66 × E-03	Ferdinandusse et al.[57] 2019
7	19117339	G	A	c.18C>T (GenBank: NM_000474.4)	*TWIST1*	Saethre-Chotzen syndrome	uStart_gain	6.92 × E-07	Diaz-Gonzalez et al.[58] 2022
7	19117576	C	T	c.255G>A (GenBank: NM_000474.4)	*TWIST1*	Saethre-Chotzen syndrome	uStart_gain	0	Zhou et al.[59] 2018
7	19117584	G	T	c.263C>A (GenBank: NM_000474.4)	*TWIST1*	Saethre-Chotzen syndrome	uStart_gain	0	Zhou et al.[59] 2018
7	117480061	C	T	c.34C>T (GenBank: NM_000492.4)	*CFTR*	disseminated bronchiectasis	uStart_gain	1.25 × E-06	Lukowski et al.[60] 2011
9	37422747	GC	AT	c.4−3delinsAT (GenBank: NM_012203.2)	*GRHPR*	primary hyperoxaluria type II	uStart_gain	0	Fu et al.[61] 2015
9	127854482	G	A	c.127C>T (GenBank: NM_001114753.3)	*ENG*	hereditary hemorrhagic telangiectasia	uStart_gain	0	Kim et al.[62] 2011
9	127854497	T	A	c.142A>T (GenBank: NM_001114753.3)	*ENG*	hereditary hemorrhagic telangiectasia	uStart_gain	0	Ruiz-Llorente et al.[63] 2019
11	299499	G	T	c.9C>A (GenBank: NM_001025295.3)	*IFITM5*	neonatal transverse clavicular fracture	uStart_gain	0	Wu et al.[64] 2020
11	299504	G	A	c.14C>T (GenBank: NM_001025295.3)	*IFITM5*	osteogenesis imperfecta, type V	uStart_gain	6.86 × E-07	Cho et al.[65] 2012
11	31806918	T	TC	c.122dup (GenBank: NM_001368894.2)	*PAX6*	aniridia	uStop_loss	0	Filatova et al.[66] 2021
11	31806921	T	TC	c.125dup (GenBank: NM_001368894.2)	*PAX6*	aniridia	uStop_loss	0	Vasilyeva et al.[67] 2017
14	67722520	C	T	c.123C>T (GenBank: NM_152443.3)	*RDH12*	macular dystrophy	uStart_gain	6.65 × E-05	Dueñas Rey et al.[68] 2024
15	66703250	G	T	c.9G>T (GenBank: NM_005585.5)	*SMAD6*	craniosynostosis	uStart_gain	3.51 × E-06	Calpena et al.[69] 2020

(*Continued on next page*)

Table 1. *Continued*

CHROM	POS	REF	ALT	HGVS	Gene	Disease	CSQ	MAF	PubMed ID Year
16	172908	CCA	C	c.3_2del (GenBank: NM_000517.6)	*HBA2*	alpha-thalassemia	mKozak	3.90 × E-06	Morlé et al.[14] 1985
16	176712	CCA	C	c.3_2del (GenBank: NM_000558.5)	*HBA1*	alpha-thalassemia	mKozak	2.49 × E-06	Viprakasit et al.[70] 2003
16	75481826	G	T	c.26C>A (GenBank: NM_021615.5)	*CHST6*	macular corneal dystrophy	uStart_gain	0	Zhang et al.[71] 2019
17	72121207	G	A	c.185G>A (GenBank: NM_000346.4)	*SOX9*	acampomelic campomelic dysplasia	uStart_gain	0	von Bohlen et al.[72] 2017
19	852326	A	T	c.3A>T (GenBank: NM_001972.4)	*ELANE*	neutropenia, severe	mKozak	0	Tidwell et al.[73] 2014
20	3084677	T	G	c.3A>C (GenBank: NM_000490.5)	*AVP*	diabetes insipidus, neurohypophyseal	mKozak	0	Ilhan et al.[74] 2016
X	67544600	C	T	c.547C>T (GenBank: NM_000044.6)	*AR*	androgen insensitivity syndrome	uStart_gain	0	Hornig et al.[75] 2016
X	68829366	C	G	c.411C>G (GenBank: NM_004429.5)	*EFNB1*	craniofrontonasal syndrome	uStart_gain	0	Romanelli Tavares et al.[76] 2019
X	68829682	T	G	c.95T>G (GenBank: NM_004429.5)	*EFNB1*	craniofrontonasal syndrome	uStop_loss	0	Twigg et al.[77] 2013
X	70133891	TAT	AA	c.[57del; 55T>A] (GenBank: NM_001551.3)	*IGBP1*	mental retardation, X-linked	uStop_gain	0	Graham et al.[78] 2003
X	151397299	C	T	c.10C>T (GenBank: NM_001017980.4)	*VMA21*	congenital disorder of glycosylation	uStart_gain	0	Cannata Serio et al.[79] 2020
Y	2787678	C	T	c.79dup (GenBank: NM_003140.3)	*SRY*	gonadal dysgenesis	uStart_gain	1.71 × E-05	Poulat et al.[80] 1997
5	102865835	G	A	c.361G>A (GenBank: NM_001177306.2)	*PAM*	pituitary adenoma	promoter	6.43 × E-03	Trivellin et al.[81] 2023
5	53109714	GGCA CAGCG GCACC ATCCC GCCTA	G	c.656−634del (GenBank: NM_004531.5)	*MOCS2*	molybdenum cofactor deficiency	start loss in another transcript	0	Hahnewald et al.[82] 2006
11	31810830	TTA	T	c.133−132del (GenBank: NM_001368894.2)	*PAX6*	aniridia	TFBS	0	Lee et al.[83] 2021
12	120978551	T	C	c.218T>C (GenBank: NM_000545.8)	*HNF1A*	diabetes, MODY	TFBS	1.14 × E-05	Godart et al.[84] 2000
X	153971810	A	G	c.970T>C (GenBank: NM_005334.3)	*HCFC1*	intellectual disability	TFBS	0	Huang et a.[85] 2012

splicing variants). The final model was trained on a dataset of 603 variants, 163 positive (HGMD) and 440 negative (gnomAD) controls, with a list of 17 features (Table S2). A threshold of 0.74 captured 90% of the positive controls. The application of this threshold to the training set yielded an accuracy of 97.5%, a sensitivity of 90.2%, and a specificity of 99.8% (Figures 4A and 4B). We assessed the robustness by 5-fold cross-validation, which showed a consistent performance (mean accuracy of 91.0% ± 4.5% at the 0.74 threshold). Feature importance analysis (Figure 5) identified uORF start codon conservation (PhyloP score; 26.5%) as the strongest predictor. Other significant features included uORF count (12.1%), gene pLI (9.8%), uORF Kozak strength (6.8%), variant effect on the uORF (5.5%), uORF proximity to 5′ end (5.4%), 5′ UTR length (4.5%), and uORF length (4.5%) as the most important. Feature ablation analysis confirmed that the model's performance was mainly driven by the specialized features unique to 5′ UTR biology, such as the uORF Kozak context strength, the count of uORF in the transcript, or the presence of ribo-seq evidence of uORF translation, rather than the generic features such as conservation alone (Figure S4). These results highlight the importance of uORF translation initiation and gene constraint for determining the impact of the variant and

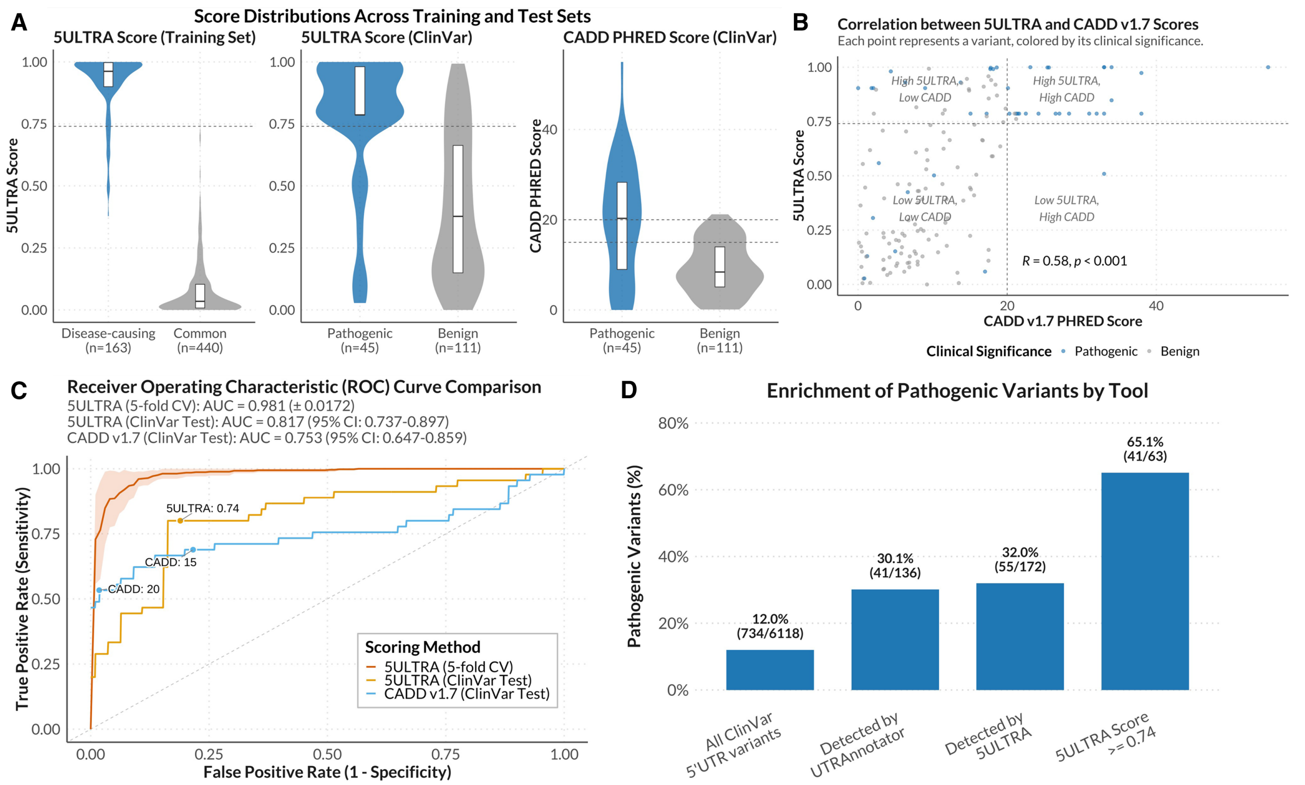

Figure 4. Evaluation of the performance of the 5ULTRA score

(A) Distributions of 5ULTRA scores for benign and pathogenic variants in the training and test datasets, and of CADD PHRED v.1.7 on the test dataset. The gray dotted line represents a threshold of 0.74 for 5ULTRA scores and 15 or 20 for the CADD scores.

(B) Scatterplot illustrating the 5ULTRA scores and the CADD scores for each variant of the test set. Blue dots are pathogenic variants. The gray dotted lines represent a threshold of 0.74 for 5ULTRA scores and 20 for the CADD scores.

(C) ROC curve comparison. Red: 5ULTRA mean from 5-fold cross-validation (CV) (mean AUC = 0.981 ± 0.017, shaded area is SD). Yellow: 5ULTRA final model applied to the independent ClinVar test set (AUC = 0.82 [0.74–0.90]). Blue: CADD v.1.7 on ClinVar (AUC = 0.75 [0.65–0.86]). Points indicate performance with thresholds of 0.74 (5ULTRA on ClinVar), and 15 or 20 (CADD).

(D) Proportions of ClinVar pathogenic variants relative to UTRAnnotator.

support the suitability of this model for evaluating new variants.

Better prioritization of pathogenic 5′ UTR variants than with CADD and UTRAnnotator

To validate the ability of the 5ULTRA score in prioritizing 5′ UTR variants affecting translation, we tested this score against existing tools on an independent ClinVar dataset,[40] from which the variants included in the training dataset were removed. There were 734 pathogenic and 5,384 benign 5′ UTR variants in the dataset, and 5ULTRA scored 156 variants as affecting uORFs (45 pathogenic and 111 benign), with a significant enrichment of pathogenic variants (odds ratio [OR] = 3.31 [2.28–4.75], $p = 8.66 \times 10^{-10}$). It should be noted that the "benign" label in ClinVar indicates a lack of disease development rather than a lack of translational impact. For these 156 variants, 5ULTRA achieved an area under the receiver-operating characteristic curve (ROC-AUC) of 0.82, vs. 0.75 for the general predictor CADD v.1.7[86] (Figure 4C), while 5ULTRA also provided a predicted translational effect and annotation. A classification threshold of 0.74 yielded an accuracy of 80.8%, with a sensitivity of 80.0% and a specificity of 81.1% (Figure S5A). With a threshold of 15, CADD achieved an accuracy of 75.6%, with a sensitivity of 68.9% and a specificity of 78.4%. With a threshold of 20, CADD achieved an accuracy of 85.3%, with a sensitivity of 53.3% and a specificity of 98.2%. We then assessed 5ULTRA against the uORF-specific tool UTRAnnotator,[27] using the 6,118 ClinVar 5′ UTR variants. The annotation of 136 variants was concordant with both tools. 5ULTRA identified an additional 36 variants (14 pathogenic), including 16 within Kozak sequences, seven affecting splicing, six long indels, five short indels, and two SNVs (Table S3). No variant was identified only by UTRAnnotator. The baseline proportions of pathogenic variants were similar (UTRAnnotator 30% vs. 5ULTRA 32%, Figure 4D), but 5ULTRA score significantly improved prioritization: the 63 variants that scored ≥0.74 were highly enriched in pathogenic variants (65% pathogenic, $p = 3.18 \times 10^{-5}$ vs. UTRAnnotator baseline, Fisher's exact test with Benjamini-Hochberg correction, Figure 4D). Thus, 5ULTRA offers both broader coverage and greater accuracy for detecting and prioritizing potentially pathogenic 5′ UTR variants affecting uORFs than CADD and UTRAnnotator.

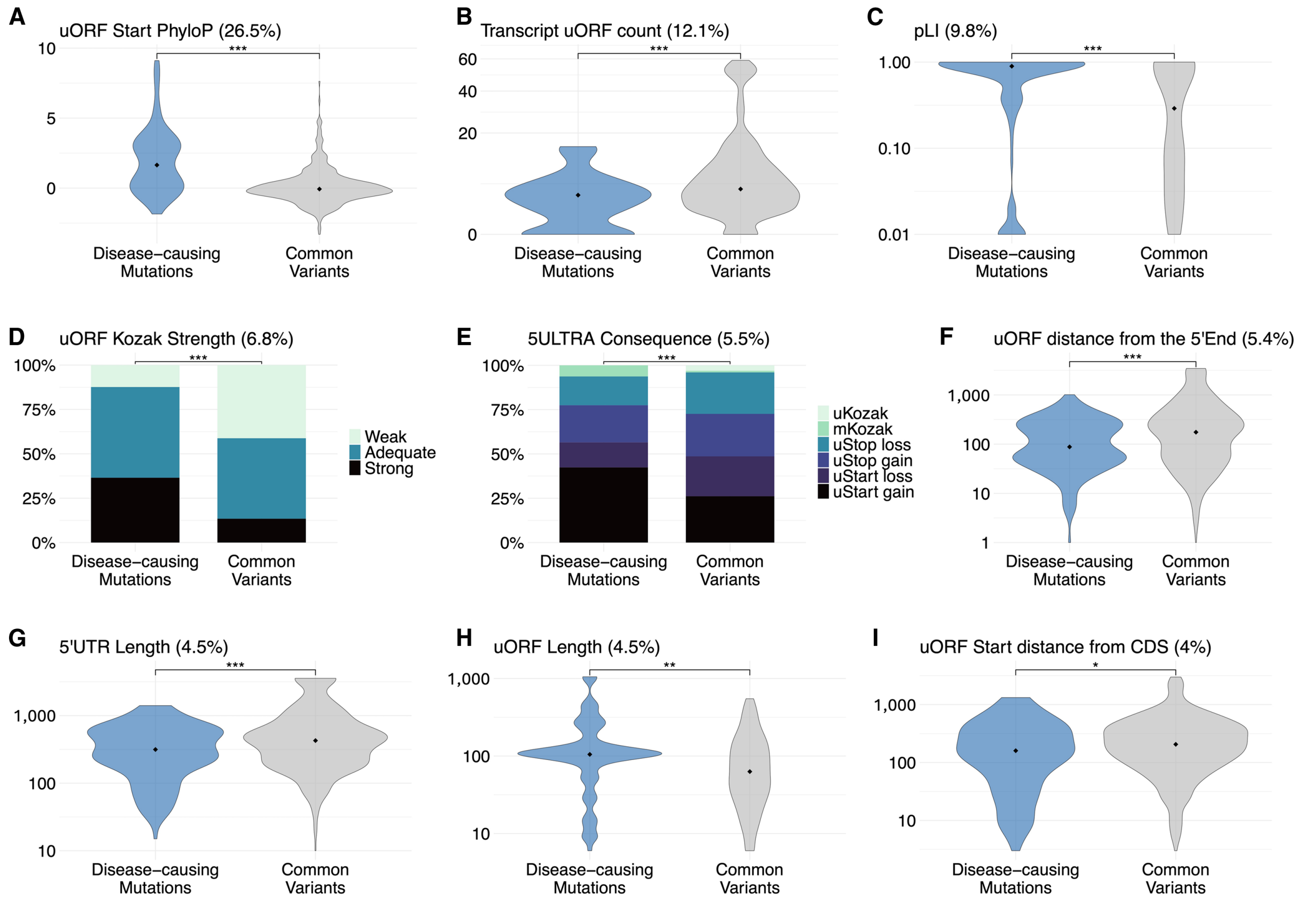

Figure 5. Feature importance analysis
Distributions of the nine top features, stratified by variant pathogenicity (disease-causing vs. common).
(A) uORF start codon PhyloP.
(B) uORF count per transcript.
(C) gene pLI.
(D) uORF Kozak strength.
(E) Variant consequence.
(F) Distance from the start of the uORF to the 5′ end (bp).
(G) 5′ UTR length (bp).
(H) uORF length (bp) (restricted to variants involving non-overlapping uORFs).
(I) Distance from the start of the uORF to the start of the CDS (bp).
Statistical tests (disease-causing vs. common): two-sample *t* tests (A, B, and E–I) and Fisher's exact tests (C and D).

5ULTRA accurately predicts the regulatory impact on protein levels

We benchmarked the performance of 5ULTRA with human proteomics data, using pQTL summary statistics[41] from the UK Biobank proteomics dataset for 46,362 individuals of inferred European genetic ancestry.[42] We restricted our analysis to 46,352 *cis*-pQTLs mapped to the 5′ UTR, defined as variants associated with protein levels at a Benjamini-Hochberg adjusted p value of <0.05 and located within the 5′ UTR of the corresponding gene. Among these, 139 were identified by 5ULTRA as variants affecting translation via uORF creation, uORF disruption, or Kozak motif disruption. Strikingly, the variants identified by 5ULTRA exerted substantially stronger effects. The median absolute effect size of 5ULTRA *cis*-pQTLs ($n = 139$) was more than five times greater than that for other 5′ UTR *cis*-pQTLs ($n =$ 46,213) (0.77 vs. 0.15; $p < 2 \times 10^{-16}$, Wilcoxon rank-sum test, Figure 6A). 5ULTRA identified variants with more significant biological consequences. Furthermore, the direction of the effect predicted by 5ULTRA (increase/decrease of protein abundance) was consistent with the observed *cis*-pQTL data ($p = 2.3 \times 10^{-9}$, Wilcoxon rank-sum test, Figure 6B). In addition, when a sign was allocated to reflect the predicted direction of the change in translation levels, the 5ULTRA score strongly and positively correlated with the experimentally measured *cis*-pQTL effect size (Spearman's correlation coefficient $= 0.57$, $p = 3.6 \times 10^{-13}$, Figure 6C). Finally, results were successfully replicated on an independent massively parallel reporter assay (MPRA) dataset[43] that captured the effect of variants on mRNA ribosome load (Spearman's correlation coefficient $= 0.781$, $p = 7.3 \times 10^{-9}$, Figure S6A).

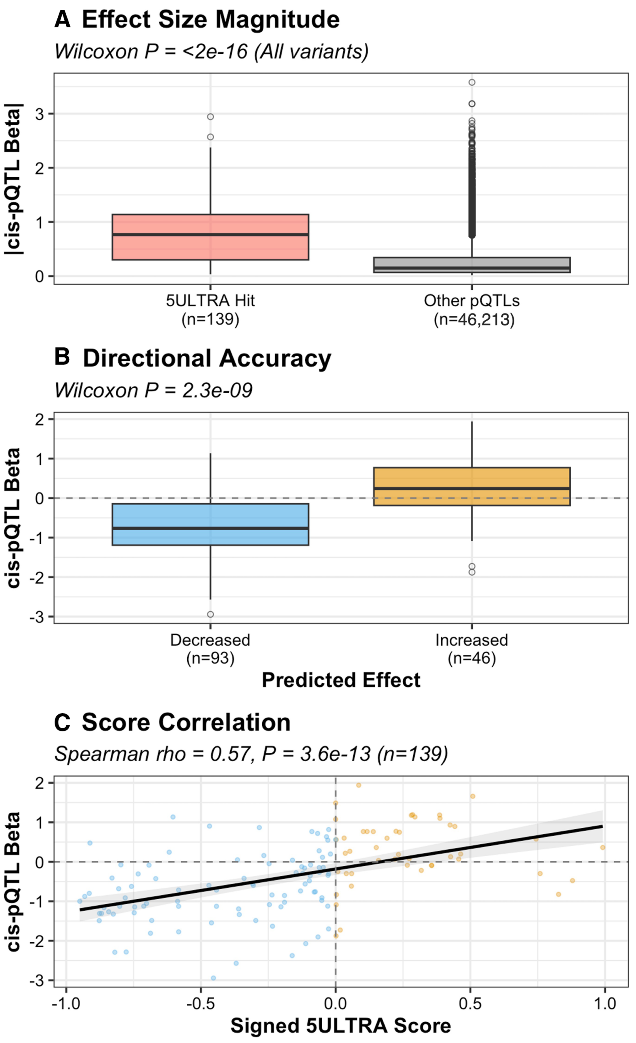

Figure 6. Validation of 5ULTRA predictions of effects on translation with FDR-controlled *cis*-pQTL data
Analysis was restricted to variants passing Benjamini-Hochberg false discovery rate control (FDR < 0.05).
(A) Comparison of absolute *cis*-pQTL effect sizes (|Beta|) between *cis*-pQTLs with a 5ULTRA prediction and other *cis*-pQTLs. The difference between the two groups is significant ($p < 2 \times 10^{-16}$, Wilcoxon rank-sum test).
(B) Comparison of *cis*-pQTL effect sizes (Beta) for variants based on the 5ULTRA-predicted effect on CDS translation ("increased" vs. "decreased"). The difference between the two groups is significant ($p = 2.3 \times 10^{-9}$, Wilcoxon rank-sum test).
(C) Correlation between 5ULTRA score (positive for predicted "increased" translation, negative for "decreased") and the *cis*-pQTL effect size (Beta) for variants with a 5ULTRA score prediction. Each point represents a variant. The line indicates the linear model fit with the 95% confidence interval shaded in gray. Significance was assessed by calculating Spearman's rank correlation coefficient (rho = 0.57, $p = 3.6 \times 10^{-13}$).

Identifying somatic 5′ UTR variants from pan-cancer data

Mutations affecting uORF have been shown to be highly prevalent in human cancers.[87,88] We screened the COSMIC database (v.99),[44] a collection of variants from 173,963 pan-cancer samples, to identify potential somatic 5′ UTR driver variants. We extracted a total of 312,241 somatic 5′ UTR variants, 19,000 (6.1%) of which were in 748 Cancer Gene Census (CGC) genes.[45] 5ULTRA predicted a total of 6,287 variants with functional consequences, 420 (6.7%) of which were in CGC genes (Table S4). Filtering by 5ULTRA score (≥0.74) resulted in 1,607 promising variants, 180 (11.2%) of which were in CGC genes. The enrichment of variants with high 5ULTRA scores among CGC genes relative to other 5′ UTR variants was significant (11.2% vs. 6.1%; $p = 6.8 \times 10^{-15}$, OR = 2.0 [1.7–2.3], Fisher's exact test, Figure S7), suggesting a potential contribution to tumorigenesis. The following examples demonstrate how 5ULTRA can be used to explain the underlying mechanisms of candidate somatic variants. 5ULTRA identified a somatic uStart-gain mutation (c.10G>T [GenBank: NM_001012750.3], absent from gnomAD, score = 0.99) in *ABI1* of a papillary renal cell carcinoma that was predicted to decrease translation of the corresponding protein significantly. ABI1 is an adaptor protein regulating actin dynamics, cell proliferation, and migration,[89] with evidence that *ABI1* played a tumor-suppressor role in gastric carcinoma.[90] Similarly, an *NRAS* 5′ UTR variant, c.17−2A>G (GenBank: NC_000001.11; NM_002524.5), absent from gnomAD, score = 0.99) was found in a breast carcinoma sample. *NRAS* is overexpressed in basal-like and HER2 tumors, the most aggressive breast cancer subtypes.[91] 5ULTRA predicted that this variant would alter splicing to convert an overlapping uORF (which has an adequate Kozak motif) into an N-terminal extension. This alteration would be expected to generate another NRAS isoform carrying this extension and to increase *NRAS* translation efficiency, possibly contributing to the high levels of the protein in aggressive tumors. The use of 5ULTRA in this context could help to identify candidate somatic variants, which could play key roles in regulating protein levels during cancer development and progression.

Common germline 5′ UTR variants significantly associated with human traits

The widespread use of GWASs has led to the discovery of numerous common genetic variants linked to human diseases and traits. However, a significant challenge remains in that many of these associations were mapped to noncoding regions, making it difficult to ascertain their functional roles. We screened the GWAS catalog database v.1.0.2,[46] attempting to establish biological explanations for some common germline 5′ UTR variants that have been shown to be significantly associated with various phenotypes (6,343 SNVs with $p < 5 \times 10^{-8}$). As 5ULTRA focuses specifically on variants altering uORFs and Kozak motifs, we used it to determine how many of these known GWAS hits might act through these mechanisms. 5ULTRA predicted 25 variants with such functional consequences (Table S5). This represents a small fraction of the variants (as other variants may affect other mechanisms, including transcription), but a significant enrichment of variants predicted by 5ULTRA was observed to the background

common 5′ UTR variants (OR = 2.39 [1.54–3.55], $p = 1.3 \times 10^{-4}$ vs. gnomAD, MAF ≥ 0.01). Four variants had scores above the threshold (≥0.74). (1) A *TAGAP* variant (c.125A>G [GenBank: NM_054114.5], score = 0.96, alternate allele frequency [AAF] = 0.52), previously lacking a mechanistic explanation despite links to multiple sclerosis,[92,93] was predicted by 5ULTRA to be a uStart-loss variant potentially increasing the levels of this T cell regulator. *TAGAP* expression is upregulated during T cell stimulation, and its levels are high in individuals with rheumatoid arthritis, another T cell-mediated autoimmune disease.[94] (2) A *VRTN* variant (c.118C>T [GenBank: NM_018228.3], score = 0.90, MAF = 0.41), associated with height and lung function,[95,96] was predicted to generate a uStop-gain in an actively translated uORF. (3) A *SPAAR* variant (c.351G>A [GenBank: NM_001348107.3], score = 0.83, MAF = 0.42), associated with cardiovascular function,[97] was predicted to create a new uORF start. (4) A *PSCA* variant (c.26C>T [GenBank: NM_005672.5], score = 0.80, MAF = 0.45) matched a known uStart-gain mechanism causing an N-terminal extension.[98] These examples demonstrate the power of 5ULTRA to reveal potential functional mechanisms for GWAS loci.

Uncovering 5′ UTR candidate variants potentially underlying susceptibility to human infectious diseases

We used 5ULTRA to screen our in-house WES/WGS database for 25,267 individuals, including individuals with severe infectious diseases and their healthy relatives. Our laboratory has recently identified a common variant significantly associated with the *Mycobacterium tuberculosis* infection resistance phenotype.[99] 5ULTRA characterized this variant of *YEATS4* (c.158C>T [GenBank: NM_006530.4], AAF = 0.66, score = 0.07) as uStart-gain, which creates a uORF attenuating *YEATS4* expression in the individuals with the T allele. A luciferase reporter assay was performed to validate this prediction. It demonstrated significantly lower levels of luciferase activity in the presence of the uORF, indicating a decrease in YEATS4 levels. We then investigated monogenic causes of severe infection by analyzing 508 known inborn errors of immunity genes[100] with 5ULTRA. This analysis yielded 601 unique variants predicted to affect protein levels, 383 of which were rare (MAF < 1%), among which 123 had high impact scores (>0.74) and 19 affected splicing. The utility of this approach can be demonstrated through two key findings. First, we validated our method by recapturing a known pathogenic splicing variant of *RPSA* (c.34+5G>C [GenBank: NC_000003.12; NM_002295.6], absent from gnomAD, score = 0.43) that has been linked to isolated congenital asplenia. [101] This variant causes partial intron retention (a 70-bp insertion confirmed by RNA sequencing) via an alternative splice site. [101] Second, we identified a potential candidate: a rare homozygous variant of *TNF* (c.171C>T [GenBank: NM_000594.4], MAF = 7×10^{-4}, score = 0.55) in an individual with tuberculosis. 5ULTRA annotated this variant as a uStart-gain variant predicted to decrease *TNF* expression. This prediction is consistent with the established links between TNF deficiency, impaired macrophage function, and susceptibility to *Mycobacterium tuberculosis* infection.[102]

Discussion

The search for disease-causing genetic variants has traditionally focused on protein-coding regions and essential splice sites. This focus has often led to an under-representation of non-coding regions, such as 5′ UTRs, in genetic analyses. However, 5′ UTR variants can profoundly affect gene expression by modulating translation initiation and efficiency, thereby playing a crucial role in disease mechanisms.[21] Our development of 5ULTRA provides a comprehensive tool for the systematic detection, annotation, and prioritization of 5′ UTR variants with a potential impact on protein translation. Appreciating the development of other tools, such as MORFEE,[25,26] UTRAnnotator,[27] and utr.annotation,[28] 5ULTRA incorporates several key advances. It includes the latest comprehensive uORF databases derived from extensive sequencing studies,[30,31] an expanded variant analysis encompassing not only SNVs and small indels but also larger variants, and, importantly, an ability to predict the functional consequences of altered splicing within multi-exon 5′ UTRs. This aspect is particularly important, as mis-splicing events can change the 5′ UTR sequence without affecting the protein-coding region while having a major effect on the regulation of translation. Furthermore, 5ULTRA provides extensive functional annotations and a machine-learning-based score validated against pQTL and MPRA datasets, features specifically designed to facilitate effective variant prioritization beyond basic annotation. 5ULTRA was benchmarked using the standardized MANE transcript set, while the tool offers flexibility to analyze all transcripts for broader discovery.

Our analysis of 28 million 5′ UTR variants from the gnomAD database with 5ULTRA revealed that variants predicted to have functional consequences are generally rarer and occur at more conserved positions than other 5′ UTR variants. This suggests that the variants detected by 5ULTRA are subject to stronger purifying selection, underscoring their potential biological importance. The 5ULTRA score—based on a random forest classifier utilizing multiple features, such as evolutionary conservation scores, Kozak sequence strength, uORF characteristics, and gene loss-of-function intolerance metrics—allows an effective prioritization of promising variants for further study. Importantly, 5ULTRA outperformed existing prediction tools in the identification of 5′ UTR variants of functional significance. Validation on an independent ClinVar dataset showed that 5ULTRA was more sensitive than CADD, with better coverage and a greater enrichment in pathogenic variants than UTRAnnotator. Furthermore,

validation with large-scale proteomics data confirmed an enrichment of 5ULTRA-identified variants among *cis*-pQTLs, with these variants exerting significantly larger effects on protein levels, 5ULTRA scores being strongly correlated with the magnitude of these effects. These results were confirmed on MPRA data measuring variants' effect on ribosome load. The predictive power of the 5ULTRA score stems from its ability to capture key biological features associated with uORF function and gene regulation. The model identified uORF start codon conservation as the most critical factor, suggesting a functional constraint on the initiation of translation. Other significant predictors, such as the number of uORFs, the intolerance of the gene to loss-of-function variants, Kozak context strength, and the direct effects of the variant on the uORF, stress the need for a comprehensive approach combining sequence-level details, evolutionary information, and gene-specific context in the evaluation of 5′ UTR variants.

Despite its strengths, 5ULTRA has limitations. A key concern is that by training on HGMD disease-causing variants vs. common gnomAD variants, the model risks learning to distinguish "rare/pathogenic vs. common" rather than "translation-impacting vs. neutral" variants. We sought to mitigate this by designing a feature set intentionally focused on translational mechanisms. The model was shown to have strong and significant correlation with experimental data (proteomics and ribosome load), providing powerful evidence that 5ULTRA is indeed capturing a true translation effect. Training on larger datasets, more ethnically diverse and with larger annotations from experimentally validated readouts, would improve the predictive power of the scoring function. 5′ UTR variants can also cause disease through other mechanisms, such as altering transcription by disrupting TFBSs located within the 5′ UTR. Moreover, 5ULTRA primarily focuses on uORFs and Kozak sequence motifs, potentially overlooking other regulatory elements within the 5′ UTR that can affect translation. These regulatory elements include non-AUG uORFs, mRNA secondary structures, internal ribosome entry sites, binding sites for RNA-binding proteins, and m6A mRNA modifications, all of which can also influence the initiation and efficiency of translation.[103–105] Other resources, such as MORFEEdb[26] and FunUV[106] already explore the impact of non-AUG uORFs and secondary structure, respectively. Incorporating annotations for these features would provide a more comprehensive assessment of 5′ UTR variants affecting translational regulation. Finally, the current model does not explicitly account for the potential interplay between multiple variants within the same 5′ UTR, which could have synergistic or antagonistic effects on translation.

5ULTRA can be applied across various domains of human genetics. In rare disease research, it facilitates the identification of pathogenic 5′ UTR variants that are typically overlooked, as demonstrated by its ability to recapture a known pathogenic splicing variant of *RPSA* causing asplenia and to identify a candidate uStart-gain variant of *TNF* in an individual susceptible to tuberculosis from our cohort. Retrospective analysis with 5ULTRA identified 37 experimentally validated pathogenic uORF or Kozak variants, demonstrating the potential of this tool to identify variants that contribute to heritable disease but are missed in traditional coding-sequence-focused analyses. In cancer genetics, 5ULTRA can identify somatic 5′ UTR variants that may act as driver mutations, increasing or decreasing the production of certain proteins, such as the candidate variants of *ABI1* and *NRAS* identified in COSMIC data, highlighting the utility of this tool for oncology research. In GWASs, 5ULTRA aids the interpretation of functional significance for non-coding variants associated with diseases or traits, providing mechanistic insights for associated variants of genes such as *TAGAP*, *VRTN*, *SPAAR*, and *PSCA*. By integrating 5ULTRA as functional weights in rare-variant association pipelines, such as STAARpipeline,[107] 5ULTRA scores can enhance the statistical power to discover gene-trait relationships in large-scale sequencing studies, thereby directly linking statistical associations with their putative functional consequences. In conclusion, 5ULTRA is a valuable resource for investigating the mechanisms by which variants affect protein translation and the genetic basis of various pathological conditions. The broad application of 5ULTRA across germline and somatic contexts, in both rare and common diseases, highlights its versatility and potential to advance our understanding of human genetics and to promote the development of precise diagnostic and therapeutic strategies for human genetic diseases.

Data and code availability

The datasets, programs, and software developed in this article are available from our HGIDSOFT webserver (https://hgidsoft.rockefeller.edu/5ULTRA) and the GitHub repository (https://github.com/casanova-lab/5ULTRA), under CC BY-NC-ND 4.0 license.

Acknowledgments

We thank Y. Nemirovskaya, D. Liu, M. Woollett, K. Francis, and L. Lorenzo for administrative support. The Laboratory of Human Genetics of Infectious Diseases is supported by the Howard Hughes Medical Institute, The Rockefeller University, the St. Giles Foundation, the National Institutes of Health (NIH) (U19AI162568), the National Center for Advancing Translational Sciences, and the NIH Clinical and Translational Science Award (CTSA) program (UL1TR001866); grants from the French National Research Agency (ANR) under France 2030 program (ANR-10-IAHU-01), the Integrative Biology of Emerging Infectious Diseases Laboratory of Excellence (ANR-10-LABX-62-IBEID), ANR GENVIR (ANR-20-CE93-003), ANR AI2D (ANR-22-CE15-0046), GENFLU (ANR-22-CE92-0004), ANR AIDIRAK (ANR-23-CE15-0011), ANR PTCRA (ANR-24-CE15-5334), ANR KDGenImmu (ANR-24-CE-1236) and ANR ILC_BY _DESIGN (ANR-24-CE-15-5475); the French Foundation for Medical Research (FRM) (EQU202503020018); the HORIZON-HLTH-2021-DISEASE-04 program under grant agreement 101057100

(UNDINE); the ANR-RHU COVIFERON program (ANR-21-RHUS-0008); the Square Foundation; Grandir - Fonds de solidarité pour l'enfance; the Fondation du Souffle; the SCOR Corporate Foundation for Science; the Battersea & Bowery Advisory Group; William E. Ford (General Atlantic's Chairman and Chief Executive Officer) and Gabriel Caillaux (General Atlantic's Co-President, Managing Director, and Head of Business in EMEA and the General Atlantic Foundation); the French Ministry of Higher Education, Research, and Innovation (MESRI-COVID-19); and INSERM, REACTing-INSERM, Paris Cité University, and the Imagine Institute. This project has received funding from the European Union's Horizon Europe research and innovation program under grant agreement no. 101156304. K.P. was supported by the David Rockefeller Graduate Program.

Declaration of interests

The authors declare no competing interests.

Supplemental information

Supplemental information can be found online at https://doi.org/10.1016/j.ajhg.2026.02.020.

Web resources

5ULTRA, https://github.com/casanova-lab/5ULTRA
GenBank, https://www.ncbi.nlm.nih.gov/genbank/
sORFs, http://www.sorfs.org

Received: August 4, 2025
Accepted: February 26, 2026
Published: March 24, 2026

References

1. Jackson, R.J., Hellen, C.U.T., and Pestova, T.V. (2010). The mechanism of eukaryotic translation initiation and principles of its regulation. Nat. Rev. Mol. Cell Biol. *11*, 113–127. https://doi.org/10.1038/nrm2838.
2. Hinnebusch, A.G. (2011). Molecular mechanism of scanning and start codon selection in eukaryotes. Microbiol. Mol. Biol. Rev. *75*, 434–467. https://doi.org/10.1128/MMBR.00008-11.
3. Kozak, M. (1981). Possible role of flanking nucleotides in recognition of the AUG initiator codon by eukaryotic ribosomes. Nucleic Acids Res. *9*, 5233–5252. https://doi.org/10.1093/nar/9.20.5233.
4. Kozak, M. (1986). Point mutations define a sequence flanking the AUG initiator codon that modulates translation by eukaryotic ribosomes. Cell *44*, 283–292. https://doi.org/10.1016/0092-8674(86)90762-2.
5. Xu, H., Wang, P., You, J., Zheng, Y., Fu, Y., Tang, Q., Zhou, L., Wei, Z., Lin, B., Shu, Y., et al. (2010). Screening of Kozak-motif-located SNPs and analysis of their association with human diseases. Biochem. Biophys. Res. Commun. *392*, 89–94. https://doi.org/10.1016/j.bbrc.2010.01.002.
6. Kozak, M. (1991). Structural features in eukaryotic mRNAs that modulate the initiation of translation. J. Biol. Chem. *266*, 19867–19870. https://doi.org/10.1016/S0021-9258(18)54860-2.
7. Ryczek, N., Łyś, A., and Makałowska, I. (2023). The Functional Meaning of 5′ UTR in Protein-Coding Genes. Int. J. Mol. Sci. *24*, 2976.
8. Morris, D.R., and Geballe, A.P. (2000). Upstream Open Reading Frames as Regulators of mRNA Translation. Mol. Cell Biol. *20*, 8635–8642.
9. Bottorff, T.A., Park, H., Geballe, A.P., and Subramaniam, A.R. (2022). Translational buffering by ribosome stalling in upstream open reading frames. PLoS Genet. *18*, e1010460. https://doi.org/10.1371/journal.pgen.1010460.
10. Whiffin, N., Karczewski, K.J., Zhang, X., Chothani, S., Smith, M.J., Evans, D.G., Roberts, A.M., Quaife, N.M., Schafer, S., Rackham, O., et al. (2020). Characterising the loss-of-function impact of 5′ untranslated region variants in 15,708 individuals. Nat. Commun. *11*, 2523. https://doi.org/10.1038/s41467-019-10717-9.
11. Zhang, P., Chaldebas, M., Ogishi, M., Al Qureshah, F., Ponsin, K., Feng, Y., Rinchai, D., Milisavljevic, B., Han, J.E., Moncada-Vélez, M., et al. (2023). Genome-wide detection of human intronic AG-gain variants located between splicing branchpoints and canonical splice acceptor sites. Proc. Natl. Acad. Sci. USA *120*, e2314225120. https://doi.org/10.1073/pnas.2314225120.
12. Zhang, P., Philippot, Q., Ren, W., Lei, W.-T., Li, J., Stenson, P.D., Palacín, P.S., Colobran, R., Boisson, B., Zhang, S.-Y., et al. (2022). Genome-wide detection of human variants that disrupt intronic branchpoints. Proc. Natl. Acad. Sci. USA *119*, e2211194119. https://doi.org/10.1073/pnas.2211194119.
13. Ellingford, J.M., Ahn, J.W., Bagnall, R.D., Baralle, D., Barton, S., Campbell, C., Downes, K., Ellard, S., Duff-Farrier, C., Fitz-Patrick, D.R., et al. (2022). Recommendations for clinical interpretation of variants found in non-coding regions of the genome. Genome Med. *14*, 73. https://doi.org/10.1186/s13073-022-01073-3.
14. Morlé, F., Lopez, B., Henni, T., and Godet, J. (1985). alpha-Thalassaemia associated with the deletion of two nucleotides at position -2 and -3 preceding the AUG codon. The EMBO journal *4*, 1245–1250. https://doi.org/10.1002/j.1460-2075.1985.tb03767.x.
15. Öner, R., Agarwal, S., Dimovski, A.J., Efremov, G.D., Petkov, G.H., Altay, C., Gurgey, A., and Huisman, T.H.J. (1991). The G→A Mutation at Position +22 31 to the Cap Site of the β-Globin Gene as a Possible Cause for a β-Thalassemia. Hemoglobin *15*, 67–76. https://doi.org/10.3109/03630269109072485.
16. Calvo, S.E., Pagliarini, D.J., and Mootha, V.K. (2009). Upstream open reading frames cause widespread reduction of protein expression and are polymorphic among humans. Proc. Natl. Acad. Sci. USA *106*, 7507–7512. https://doi.org/10.1073/pnas.0810916106.
17. Lee, D.S.M., Park, J., Kromer, A., Baras, A., Rader, D.J., Ritchie, M.D., Ghanem, L.R., and Barash, Y. (2021). Disrupting upstream translation in mRNAs is associated with human disease. Nat. Commun. *12*, 1515. https://doi.org/10.1038/s41467-021-21812-1.
18. Grant, C.M., and Hinnebusch, A.G. (1994). Effect of sequence context at stop codons on efficiency of reinitiation in GCN4 translational control. Mol. Cell Biol. *14*, 606–618. https://doi.org/10.1128/mcb.14.1.606-618.1994.
19. Tanaka, M., Sotta, N., Yamazumi, Y., Yamashita, Y., Miwa, K., Murota, K., Chiba, Y., Hirai, M.Y., Akiyama, T., Onouchi,

H., et al. (2016). The Minimum Open Reading Frame, AUG-Stop, Induces Boron-Dependent Ribosome Stalling and mRNA Degradation. Plant Cell *28*, 2830–2849. https://doi.org/10.1105/tpc.16.00481.

20. Dasgupta, A., and Prensner, J.R. (2024). Upstream open reading frames: new players in the landscape of cancer gene regulation. NAR Cancer *6*, zcae023. https://doi.org/10.1093/narcan/zcae023.
21. Chatterjee, S., and Pal, J.K. (2009). Role of 5′- and 3′-untranslated regions of mRNAs in human diseases. Biol. Cell *101*, 251–262. https://doi.org/10.1042/BC20080104.
22. Wieder, N., D'Souza, E.N., Martin-Geary, A.C., Lassen, F.H., Talbot-Martin, J., Fernandes, M., Chothani, S.P., Rackham, O.J.L., Schafer, S., Aspden, J.L., et al. (2024). Differences in 5′untranslated regions highlight the importance of translational regulation of dosage sensitive genes. Genome Biol. *25*, 111. https://doi.org/10.1186/s13059-024-03248-0.
23. Chatterjee, S., Rao, S.J., and Pal, J.K. (2017). Pathological mutations in 5′ untranslated regions of human genes. In Encyclopedia of Life Sciences (eLS). https://doi.org/10.1002/9780470015902.a0022408.pub2.
24. Bohn, E., Lau, T.T.Y., Wagih, O., Masud, T., and Merico, D. (2023). A curated census of pathogenic and likely pathogenic UTR variants and evaluation of deep learning models for variant effect prediction. Front. Mol. Biosci. *10*, 1257550. https://doi.org/10.3389/fmolb.2023.1257550.
25. Aïssi, D., Soukarieh, O., Proust, C., Jaspard-Vinassa, B., Fautrad, P., Ibrahim-Kosta, M., Leal-Valentim, F., Roux, M., Bacq-Daian, D., Olaso, R., et al. (2020). MORFEE: a new tool for detecting and annotating single nucleotide variants creating premature ATG codons from VCF files. bioRxiv. https://doi.org/10.1101/2020.03.29.012054.
26. Meguerditchian, C., Baux, D., Ludwig, T.E., Genin, E., Trégouët, D.-A., and Soukarieh, O. (2025). Enhancing the annotation of small ORF-altering variants using MORFEE: introducing MORFEEdb, a comprehensive catalog of SNVs affecting upstream ORFs in human 5′UTRs. NAR Genom. Bioinform. *7*, lqaf017. https://doi.org/10.1093/nargab/lqaf017.
27. Zhang, X., Wakeling, M., Ware, J., and Whiffin, N. (2021). Annotating high-impact 5′untranslated region variants with the UTRannotator. Bioinformatics *37*, 1171–1173. https://doi.org/10.1093/bioinformatics/btaa783.
28. Liu, Y., and Dougherty, J.D. (2021). utr.annotation: a tool for annotating genomic variants that could influence post-transcriptional regulation. Bioinformatics *37*, 3926–3928. https://doi.org/10.1093/bioinformatics/btab635.
29. Olexiouk, V., Crappé, J., Verbruggen, S., Verhegen, K., Martens, L., and Menschaert, G. (2016). sORFs.org: a repository of small ORFs identified by ribosome profiling. Nucleic Acids Res. *44*, D324–D329. https://doi.org/10.1093/nar/gkv1175.
30. Manske, F., Ogoniak, L., Jürgens, L., Grundmann, N., Makałowski, W., and Wethmar, K. (2023). The new uORFdb: integrating literature, sequence, and variation data in a central hub for uORF research. Nucleic Acids Res. *51*, D328–D336. https://doi.org/10.1093/nar/gkac899.
31. Liu, Q., Peng, X., Shen, M., Qian, Q., Xing, J., Li, C., and Gregory, R.I. (2023). Ribo-uORF: a comprehensive data resource of upstream open reading frames (uORFs) based on ribosome profiling. Nucleic Acids Res. *51*, D248–D261. https://doi.org/10.1093/nar/gkac1094.
32. Iacono, M., Mignone, F., and Pesole, G. (2005). uAUG and uORFs in human and rodent 5′untranslated mRNAs. Gene *349*, 97–105. https://doi.org/10.1016/j.gene.2004.11.041.
33. Frankish, A., Carbonell-Sala, S., Diekhans, M., Jungreis, I., Loveland, J.E., Mudge, J.M., Sisu, C., Wright, J.C., Arnan, C., Barnes, I., et al. (2023). GENCODE: reference annotation for the human and mouse genomes in 2023. Nucleic Acids Res. *51*, D942–D949. https://doi.org/10.1093/nar/gkac1071.
34. Rausell, A., Luo, Y., Lopez, M., Seeleuthner, Y., Rapaport, F., Favier, A., Stenson, P.D., Cooper, D.N., Patin, E., Casanova, J.-L., et al. (2020). Common homozygosity for predicted loss-of-function variants reveals both redundant and advantageous effects of dispensable human genes. Proc. Natl. Acad. Sci. USA *117*, 13626–13636. https://doi.org/10.1073/pnas.1917993117.
35. Jaganathan, K., Kyriazopoulou Panagiotopoulou, S., McRae, J.F., Darbandi, S.F., Knowles, D., Li, Y.I., Kosmicki, J.A., Arbelaez, J., Cui, W., Schwartz, G.B., et al. (2019). Predicting Splicing from Primary Sequence with Deep Learning. Cell *176*, 535–548.e24. https://doi.org/10.1016/j.cell.2018.12.015.
36. Chen, S., Francioli, L.C., Goodrich, J.K., Collins, R.L., Kanai, M., Wang, Q., Alföldi, J., Watts, N.A., Vittal, C., Gauthier, L.D., et al. (2024). A genomic mutational constraint map using variation in 76,156 human genomes. Nature *625*, 92–100. https://doi.org/10.1038/s41586-023-06045-0.
37. Kuhn, R.M., Haussler, D., and Kent, W.J. (2013). The UCSC genome browser and associated tools. Brief. Bioinform. *14*, 144–161. https://doi.org/10.1093/bib/bbs038.
38. Pollard, K.S., Hubisz, M.J., Rosenbloom, K.R., and Siepel, A. (2010). Detection of nonneutral substitution rates on mammalian phylogenies. Genome Res. *20*, 110–121. https://doi.org/10.1101/gr.097857.109.
39. Stenson, P.D., Mort, M., Ball, E.V., Chapman, M., Evans, K., Azevedo, L., Hayden, M., Heywood, S., Millar, D.S., Phillips, A.D., and Cooper, D.N. (2020). The Human Gene Mutation Database (HGMD®): optimizing its use in a clinical diagnostic or research setting. Hum. Genet. *139*, 1197–1207. https://doi.org/10.1007/s00439-020-02199-3.
40. Landrum, M.J., Lee, J.M., Riley, G.R., Jang, W., Rubinstein, W.S., Church, D.M., and Maglott, D.R. (2014). ClinVar: public archive of relationships among sequence variation and human phenotype. Nucleic Acids Res. *42*, D980–D985. https://doi.org/10.1093/nar/gkt1113.
41. Hawkes, G., Chundru, K., Jackson, L., Patel, K.A., Murray, A., Wood, A.R., Wright, C.F., Weedon, M.N., Frayling, T.M., and Beaumont, R.N. (2025). Whole-genome sequencing analysis identifies rare, large-effect noncoding variants and regulatory regions associated with circulating protein levels. Nat. Genet. *57*, 626–634. https://doi.org/10.1038/s41588-025-02095-4.
42. Sudlow, C., Gallacher, J., Allen, N., Beral, V., Burton, P., Danesh, J., Downey, P., Elliott, P., Green, J., Landray, M., et al. (2015). UK biobank: an open access resource for identifying the causes of a wide range of complex diseases of middle and old age. PLoS Med. *12*, e1001779. https://doi.org/10.1371/journal.pmed.1001779.
43. Sample, P.J., Wang, B., Reid, D.W., Presnyak, V., McFadyen, I.J., Morris, D.R., and Seelig, G. (2019). Human 5′ UTR design and variant effect prediction from a massively parallel translation assay. Nat. Biotechnol. *37*, 803–809. https://doi.org/10.1038/s41587-019-0164-5.

44. Sondka, Z., Dhir, N.B., Carvalho-Silva, D., Jupe, S., Ahmed, M., McLaren, K., McLaren, K., Starkey, M., Ward, S., Wilding, J., et al. (2024). COSMIC: a curated database of somatic variants and clinical data for cancer. Nucleic Acids Res. *52*, D1210–D1217. https://doi.org/10.1093/nar/gkad986.
45. Sondka, Z., Bamford, S., Cole, C.G., Ward, S.A., Dunham, I., and Forbes, S.A. (2018). The COSMIC Cancer Gene Census: describing genetic dysfunction across all human cancers. Nat. Rev. Cancer *18*, 696–705. https://doi.org/10.1038/s41568-018-0060-1.
46. Sollis, E., Mosaku, A., Abid, A., Buniello, A., Cerezo, M., Gil, L., Groza, T., Güneş, O., Hall, P., Hayhurst, J., et al. (2023). The NHGRI-EBI GWAS Catalog: knowledgebase and deposition resource. Nucleic Acids Res. *51*, D977–D985. https://doi.org/10.1093/nar/gkac1010.
47. Morales, J., Pujar, S., Loveland, J.E., Astashyn, A., Bennett, R., Berry, A., Cox, E., Davidson, C., Ermolaeva, O., Farrell, C.M., et al. (2022). A joint NCBI and EMBL-EBI transcript set for clinical genomics and research. Nature *604*, 310–315. https://doi.org/10.1038/s41586-022-04558-8.
48. Ho, T.K. (1995). Random decision forests. In Proceedings of 3rd International Conference on Document Analysis and Recognition (IEEE), pp. 278–282.
49. Willemsen, M.A., Vissers, L.E., Verbeek, M.M., van Bon, B.W., Geuer, S., Gilissen, C., Klepper, J., Kwint, M.P., Leen, W.G., Pennings, M., Wevers, R.A., Veltman, J.A., and Kamsteeg, E.J. (2017). Upstream SLC2A1 translation initiation causes GLUT1 deficiency syndrome. Eur. J. Hum. Genet. *25*, 771–774. 6. https://doi.org/10.1038/ejhg.2017.45.
50. Krude, H., Biebermann, H., Luck, W., Horn, R., Brabant, G., and Grüters, A. (1998). Severe early-onset obesity, adrenal insufficiency and red hair pigmentation caused by POMC mutations in humans. Nat. Genet. *19*, 155–157. 2. https://doi.org/10.1038/509.
51. Aldred, M.A., Machado, R.D., James, V., Morrell, N.W., and Trembath, R.C. (2007). Characterization of the BMPR2 5'-untranslated region and a novel mutation in pulmonary hypertension. Am. J. Respir. Crit. Care Med. *176*, 819–824. 8. https://doi.org/10.1164/rccm.200701-164OC.
52. Labrouche-Colomer, S., Soukarieh, O., Proust, C., Mouton, C., Huguenin, Y., Roux, M., Besse, C., Boland, A., Olaso, R., Constans, J., Deleuze, J.F., Morange, P.E., Jaspard-Vinassa, B., Trégouët, D.A.; and GenMed Consortium (2020). GenMed Consortium. A novel rare c.-39C>T mutation in the PROS1 5'UTR causing PS deficiency by creating a new upstream translation initiation codon. Clin. Sci. *134*, 1181–1190. 10. https://doi.org/10.1042/CS20200403.
53. Ghilardi, N., Wiestner, A., Kikuchi, M., Ohsaka, A., and Skoda, R.C. (1999). Hereditary thrombocythaemia in a Japanese family is caused by a novel point mutation in the thrombopoietin gene. Br. J. Haematol. *107*, 310. 2. https://doi.org/10.1046/j.1365-2141.1999.01710.x.
54. Pendleton, A., Johnson, M.D., Hughes, A., Gurley, K.A., Ho, A.M., Doherty, M., Dixey, J., Gillet, P., Loeuille, D., McGrath, R., et al. (2002). Mutations in ANKH cause chondrocalcinosis. Am. J. Hum. Genet. *71*, 933. 4. https://doi.org/10.1086/343054.
55. Coursimault, J., Rovelet-Lecrux, A., Cassinari, K., Brischoux-Boucher, E., Saugier-Veber, P., Goldenberg, A., Lecoquierre, F., Drouot, N., Richard, A.C., Vera, G., et al. (2022). uORF-introducing variants in the 5'UTR of the NIPBL gene as a cause of Cornelia de Lange syndrome. Hum. Mutat. *43*, 1239–1248. 9. https://doi.org/10.1002/humu.24384.
56. Wright, C.F., Quaife, N.M., Ramos-Hernández, L., Danecek, P., Ferla, M.P., Samocha, K.E., Kaplanis, J., Gardner, E.J., Eberhardt, R.Y., Chao, K.R., et al. (2021). Non-coding region variants upstream of MEF2C cause severe developmental disorder through three distinct loss-of-function mechanisms. Am J Hum Genet. *108*, 1083–1094. 6. https://doi.org/10.1016/j.ajhg.2021.04.025.
57. Ferdinandusse, S., Te Brinke, H., Ruiter, J.P.N., Haasjes, J., Oostheim, W., van Lenthe, H., IJlst, L., Ebberink, M.S., Wanders, R.J.A., Vaz, F.M., et al. (2019). A mutation creating an upstream translation initiation codon in SLC22A5 5'UTR is a frequent cause of primary carnitine deficiency. Hum. Mutat. *40*, 1899–1904. 10. https://doi.org/10.1002/humu.23839.
58. Diaz-Gonzalez, F., Sacedo-Gutiérrez, J.M., Twigg, S.R.F., Calpena, E., Carceller-Benito, F.E., Parrón-Pajares, M., Santos-Simarro, F., and Heath, K.E. (2023). Case report: A third variant in the 5' UTR of TWIST1 creates a novel upstream translation initiation site in a child with Saethre-Chotzen syndrome. Front Genet. *13*, 1089417. https://doi.org/10.3389/fgene.2022.1089417.
59. Zhou, Y., Koelling, N., Fenwick, A.L., McGowan, S.J., Calpena, E., Wall, S.A., Smithson, S.F., Wilkie, A.O.M., and Twigg, S.R.F. (2018). Disruption of TWIST1 translation by 5' UTR variants in Saethre-Chotzen syndrome. Hum. Mutat. *39*, 1360–1365. 10. https://doi.org/10.1002/humu.23598.
60. Lukowski, S.W., Bombieri, C., and Trezise, A.E.O. (2011). Disrupted post-transcriptional regulation of the cystic fibrosis transmembrane conductance regulator (CFTR) by a 5'UTR mutation is associated with a CFTR-related disease. Hum. Mutat. *32*, E2266. 10. https://doi.org/10.1002/humu.21545.
61. Fu, Y., Rope, R., Fargue, S., Cohen, H.T., Holmes, R.P., and Cohen, D.M. (2015). A mutation creating an out-of-frame alternative translation initiation site in the GRHPR 5'UTR causing primary hyperoxaluria type II. Clin. Genet. *88*, 494. 5. https://doi.org/10.1111/cge.12541.
62. Kim, M.J., Kim, S.T., Lee, H.D., Lee, K.Y., Seo, J., Lee, J.B., Lee, Y.J., and Oh, S.P. (2011). Clinical and genetic analyses of three Korean families with hereditary hemorrhagic telangiectasia. BMC Med. Genet. *12*, 130. https://doi.org/10.1186/1471-2350-12-130.
63. Ruiz-Llorente, L., McDonald, J., Wooderchak-Donahue, W., Briggs, E., Chesnutt, M., Bayrak-Toydemir, P., and Bernabeu, C. (2019). Characterization of a family mutation in the 5' untranslated region of the endoglin gene causative of hereditary hemorrhagic telangiectasia. J. Hum. Genet. *64*, 333–339. 4. https://doi.org/10.1038/s10038-019-0564-x.
64. Wu, D., Wang, Y., and Huang, H. (2020). A novel variant of the IFITM5 gene within the 5'-UTR causes neonatal transverse clavicular fracture: Expanding the genetic spectrum. Mol. Genet. Genomic Med. *8*, e1287. 7. https://doi.org/10.1002/mgg3.1287.
65. Cho, T.J., Lee, K.E., Lee, S.K., Song, S.J., Kim, K.J., Jeon, D., Lee, G., Kim, H.N., Lee, H.R., Eom, H.H., et al. (2012). A single recurrent mutation in the 5'-UTR of IFITM5 causes osteogenesis imperfecta type V. Am. J. Hum. Genet. *91*, 343. 2. https://doi.org/10.1016/j.ajhg.2012.06.005.
66. Filatova, A.Y., Vasilyeva, T.A., Marakhonov, A.V., Sukhanova, N.V., Voskresenskaya, A.A., Zinchenko, R.A., and

Skoblov, M.Y. (2021). Upstream ORF frameshift variants in the PAX6 5'UTR cause congenital aniridia. Hum. Mutat. *42*, 1053–1065. 8. https://doi.org/10.1002/humu.24248.

67. Vasilyeva, T.A., Voskresenskaya, A.A., Käsmann-Kellner, B., Khlebnikova, O.V., Pozdeyeva, N.A., Bayazutdinova, G.M., Kutsev, S.I., Ginter, E.K., Semina, E.V., Marakhonov, A.V., et al. (2017). Molecular analysis of patients with aniridia in Russian Federation broadens the spectrum of PAX6 mutations. Clin. Genet. *92*, 639–644. 6. https://doi.org/10.1111/cge.13019.
68. Dueñas Rey, A., Del Pozo Valero, M., Bouckaert, M., Wood, K.A., Van den Broeck, F., Daich Varela, M., Thomas, H.B., Van Heetvelde, M., De Bruyne, M., Van de Sompele, S.,B., et al. (2024). Combining a prioritization strategy and functional studies nominates 5'UTR variants underlying inherited retinal disease. Genome Med *16*, 7. 1. https://doi.org/10.1186/s13073-023-01277-1.
69. Calpena, E., Cuellar, A., Bala, K., Swagemakers, S.M.A., Koelling, N., McGowan, S.J., Phipps, J.M., Balasubramanian, M., Cunningham, M.L., Douzgou, S., et al. (2020). Genomics England Research Consortium; Boyadjiev SA, Wilkie AOM. SMAD6 variants in craniosynostosis: genotype and phenotype evaluation. Genet. Med. *22*, 1498–1506. 9. https://doi.org/10.1038/s41436-020-0817-2.
70. Viprakasit, V., Ayyub, H., and May, A. (2003). Dinucleotide deletion in -alpha3.7 allele causes a severe form of alpha+ thalassaemia. Eur. J. Haematol. *71*, 133. 2. https://doi.org/10.1034/j.1600-0609.2003.00106.x.
71. Zhang, W., Kassels, A.C., Barrington, A., Khan, S., Tomatsu, S., Alkadi, T., and Aldave, A. (2019). Macular corneal dystrophy with isolated peripheral Descemet membrane deposits. Am. J. Ophthalmol. Case Rep. *16*, 100571. https://doi.org/10.1016/j.ajoc.2019.100571.
72. von Bohlen, A.E., Böhm, J., Pop, R., Johnson, D.S., Tolmie, J., Stücker, R., Morris-Rosendahl, D., and Scherer, G. (2017). A mutation creating an upstream initiation codon in the SOX9 5' UTR causes acampomelic campomelic dysplasia. Mol. Genet. Genomic Med. *5*, 261–268. 3. https://doi.org/10.1002/mgg3.282.
73. Tidwell, T., Wechsler, J., Nayak, R.C., Trump, L., Salipante, S.J., Cheng, J.C., Donadieu, J., Glaubach, T., Corey, S.J., Grimes, H.L., et al. (2014). Neutropenia-associated ELANE mutations disrupting translation initiation produce novel neutrophil elastase isoforms. Blood *123*, 562. 4. https://doi.org/10.1182/blood-2013-07-513242.
74. Ilhan, M., Tiryakioglu, N.O., Karaman, O., Coskunpinar, E., Yildiz, R.S., Turgut, S., Tiryakioglu, D., Toprak, H., and Tasan, E. (2016). A novel AVP gene mutation in a Turkish family with neurohypophyseal diabetes insipidus. J. Endocrinol. Invest. *39*, 285–290. 3. https://doi.org/10.1007/s40618-015-0357-9.
75. Hornig, N.C., de Beaufort, C., Denzer, F., Cools, M., Wabitsch, M., Ukat, M., Kulle, A.E., Schweikert, H.U., Werner, R., Hiort, O., et al. (2016). A Recurrent Germline Mutation in the 5'UTR of the Androgen Receptor Causes Complete Androgen Insensitivity by Activating Aberrant uORF Translation. PLoS One *11*, e0154158. https://doi.org/10.1371/journal.pone.0154158.
76. Romanelli Tavares, V.L., Kague, E., Musso, C.M., Alegria, T.G.P., Freitas, R.S., Bertola, D.R., Twigg, S.R.F., and Passos-Bueno, M.R. (2019). Craniofrontonasal Syndrome Caused by Introduction of a Novel uATG in the 5'UTR of EFNB1. Mol. Syndromol. *10*, 40–47. 1-2. https://doi.org/10.1159/000490635.
77. Twigg, S.R.F., Babbs, C., van den Elzen, M.E.P., Goriely, A., Taylor, S., McGowan, S.J., Giannoulatou, E., Lonie, L., Ragoussis, J., Sadighi Akha, E., et al. (2013). Cellular interference in craniofrontonasal syndrome: males mosaic for mutations in the X-linked EFNB1 gene are more severely affected than true hemizygotes. Hum. Mol. Genet. *22*, 1654. 8. https://doi.org/10.1093/hmg/ddt015.
78. Graham, J.M., Jr., Wheeler, P., Tackels-Horne, D., Lin, A.E., Hall, B.D., May, M., Short, K.M., Schwartz, C.E., and Cox, T.C. (2003). A new X-linked syndrome with agenesis of the corpus callosum, mental retardation, coloboma, micrognathia, and a mutation in the Alpha 4 gene at Xq13. Am. J. Med. Genet. A *123A*, 37–44. 1. https://doi.org/10.1002/ajmg.a.20504.
79. Cannata Serio, M., Graham, L.A., Ashikov, A., Larsen, L.E., Raymond, K., Timal, S., Le Meur, G., Ryan, M., Czarnowska, E., Jansen, J.C., et al. (2020). Mutations in the V-ATPase Assembly Factor VMA21 Cause a Congenital Disorder of Glycosylation With Autophagic Liver Disease. Hepatology *72*, 1968–1986. 6. https://doi.org/10.1002/hep.31218.
80. Poulat, F., Desclozeaux, M., Tuffery, S., Jay, P., Boizet, B., and Berta, P. (1998). Mutation in the 5' noncoding region of the SRY gene in an XY sex-reversed patient. Hum Mutat *1*, S192. https://doi.org/10.1002/humu.1380110162.
81. Trivellin, G., Daly, A.F., Hernández-Ramírez, L.C., Araldi, E., Tatsi, C., Dale, R.K., Fridell, G., Mittal, A., Faucz, F.R., Iben, J.R., et al. (2023). Germline loss-of-function PAM variants are enriched in subjects with pituitary hypersecretion. Front. Endocrinol. *14*, 1166076. https://doi.org/10.3389/fendo.2023.1166076.
82. Hahnewald, R., Leimkühler, S., Vilaseca, A., Acquaviva-Bourdain, C., Lenz, U., and Reiss, J. (2006). A novel MOCS2 mutation reveals coordinated expression of the small and large subunit of molybdopterin synthase. Mol. Genet. Metab. *89*, 210. 3. https://doi.org/10.1016/j.ymgme.2006.04.008.
83. Lee, J., Suh, Y., Jeong, H., Kim, G.H., Byeon, S.H., Han, J., and Lim, H.T. (2021). Aberrant expression of PAX6 gene associated with classical aniridia: identification and functional characterization of novel noncoding mutations. J. Hum. Genet. *66*, 333–338. 3. https://doi.org/10.1038/s10038-020-00829-2.
84. Godart, F., Bellanné-Chantelot, C., Clauin, S., Gragnoli, C., Abderrahmani, A., Blanché, H., Boutin, P., Chèvre, J.C., Froguel, P., and Bailleul, B. (2000). Identification of seven novel nucleotide variants in the hepatocyte nuclear factor-1alpha (TCF1) promoter region in MODY patients. Hum Mutat *15*, 173. 2. https://doi.org/10.1002/(SICI)1098-1004(200002)15:2<173::AID-HUMU6>3.0.CO;2-W.
85. Huang, L., Jolly, L.A., Willis-Owen, S., Gardner, A., Kumar, R., Douglas, E., Shoubridge, C., Wieczorek, D., Tzschach, A., Cohen, M., et al. (2012). A noncoding, regulatory mutation implicates HCFC1 in nonsyndromic intellectual disability. Am. J. Hum. Genet. *91*, 694–702. 4. https://doi.org/10.1016/j.ajhg.2012.08.011.
86. Schubach, M., Maass, T., Nazaretyan, L., Röner, S., and Kircher, M. (2024). CADD v1.7: using protein language models, regulatory CNNs and other nucleotide-level scores to improve genome-wide variant predictions. Nucleic Acids Res. *52*, D1143–D1154. https://doi.org/10.1093/nar/gkad989.

87. Jürgens, L., Manske, F., Hubert, E., Kischka, T., Flötotto, L., Klaas, O., Shabardina, V., Schliemann, C., Makalowski, W., and Wethmar, K. (2021). Somatic Functional Deletions of Upstream Open Reading Frame-Associated Initiation and Termination Codons in Human Cancer. Biomedicines 9, 618.
88. Schulz, J., Mah, N., Neuenschwander, M., Kischka, T., Ratei, R., Schlag, P.M., Castaños-Vélez, E., Fichtner, I., Tunn, P.-U., Denkert, C., et al. (2018). Loss-of-function uORF mutations in human malignancies. Sci. Rep. *8*, 2395. https://doi.org/10.1038/s41598-018-19201-8.
89. Dubielecka, P.M., Ladwein, K.I., Xiong, X., Migeotte, I., Chorzalska, A., Anderson, K.V., Sawicki, J.A., Rottner, K., Stradal, T.E., and Kotula, L. (2011). Essential role for Abi1 in embryonic survival and WAVE2 complex integrity. Proc. Natl. Acad. Sci. USA *108*, 7022–7027. https://doi.org/10.1073/pnas.1016811108.
90. Cui, M., Yu, W., Dong, J., Chen, J., Zhang, X., and Liu, Y. (2010). Downregulation of ABI1 expression affects the progression and prognosis of human gastric carcinoma. Med. Oncol. *27*, 632–639. https://doi.org/10.1007/s12032-009-9260-6.
91. Galiè, M. (2019). RAS as Supporting Actor in Breast Cancer. Front. Oncol. *9*, 2019. https://doi.org/10.3389/fonc.2019.01199.
92. Consortium*†, I.M.S.G., Patsopoulos, N.A., Baranzini, S.E., Santaniello, A., Shoostari, P., Cotsapas, C., Wong, G., Beecham, A.H., James, T., Replogle, J., et al. (2019). Multiple sclerosis genomic map implicates peripheral immune cells and microglia in susceptibility. Science *365*, eaav7188. https://doi.org/10.1126/science.aav7188.
93. Chen, M.-H., Raffield, L.M., Mousas, A., Sakaue, S., Huffman, J.E., Moscati, A., Trivedi, B., Jiang, T., Akbari, P., Vuckovic, D., et al. (2020). Trans-ethnic and Ancestry-Specific Blood-Cell Genetics in 746,667 Individuals from 5 Global Populations. Cell *182*, 1198–1213.e14. https://doi.org/10.1016/j.cell.2020.06.045.
94. Arshad, M., Bhatti, A., John, P., Jalil, F., Borghese, F., Kawalkowska, J.Z., Williams, R.O., and Clanchy, F.I.L. (2018). T cell activation Rho GTPase activating protein (TAGAP) is upregulated in clinical and experimental arthritis. Cytokine *104*, 130–135. https://doi.org/10.1016/j.cyto.2017.10.002.
95. Yengo, L., Vedantam, S., Marouli, E., Sidorenko, J., Bartell, E., Sakaue, S., Graff, M., Eliasen, A.U., Jiang, Y., Raghavan, S., et al. (2022). A saturated map of common genetic variants associated with human height. Nature *610*, 704–712. https://doi.org/10.1038/s41586-022-05275-y.
96. Shrine, N., Izquierdo, A.G., Chen, J., Packer, R., Hall, R.J., Guyatt, A.L., Batini, C., Thompson, R.J., Pavuluri, C., Malik, V., et al. (2023). Multi-ancestry genome-wide association analyses improve resolution of genes and pathways influencing lung function and chronic obstructive pulmonary disease risk. Nat. Genet. *55*, 410–422. https://doi.org/10.1038/s41588-023-01314-0.
97. Eppinga, R.N., Hagemeijer, Y., Burgess, S., Hinds, D.A., Stefansson, K., Gudbjartsson, D.F., van Veldhuisen, D.J., Munroe, P.B., Verweij, N., and van der Harst, P. (2016). Identification of genomic loci associated with resting heart rate and shared genetic predictors with all-cause mortality. Nat. Genet. *48*, 1557–1563. https://doi.org/10.1038/ng.3708.
98. Wu, X., Ye, Y., Kiemeney, L.A., Sulem, P., Rafnar, T., Matullo, G., Seminara, D., Yoshida, T., Saeki, N., Andrew, A.S., et al. (2009). Genetic variation in the prostate stem cell antigen gene PSCA confers susceptibility to urinary bladder cancer. Nat. Genet. *41*, 991–995. https://doi.org/10.1038/ng.421.
99. Conil, C., Bohlen, J., Kroon, E.E., Jean-Juste, M.A., Manry, J., Chaldebas, M., Bean, J.M., Walsh, K.F., Dallmann-Sauer, M., Rotival, M., et al. (2025). A human YEATS4 variant confers resistance to TST and IGRA conversion despite Mycobacterium tuberculosis exposure. Genome Med. *17*, 121. https://doi.org/10.1186/s13073-025-01547-0.
100. Poli, M.C., Aksentijevich, I., Bousfiha, A.A., Cunningham-Rundles, C., Hambleton, S., Klein, C., Morio, T., Picard, C., Puel, A., Rezaei, N., et al. (2025). Human inborn errors of immunity: 2024 update on the classification from the International Union of Immunological Societies Expert Committee. J. Hum. Immun. *1*, e20250003. https://doi.org/10.70962/jhi.20250003.
101. Bolze, A., Boisson, B., Bosch, B., Antipenko, A., Bouaziz, M., Sackstein, P., Chaker-Margot, M., Barlogis, V., Briggs, T., Colino, E., et al. (2018). Incomplete penetrance for isolated congenital asplenia in humans with mutations in translated and untranslated RPSA exons. Proc. Natl. Acad. Sci. USA *115*, E8007–E8016. https://doi.org/10.1073/pnas.1805437115.
102. Arias, A.A., Neehus, A.-L., Ogishi, M., Meynier, V., Krebs, A., Lazarov, T., Lee, A.M., Arango-Franco, C.A., Yang, R., Orrego, J., et al. (2024). Tuberculosis in otherwise healthy adults with inherited TNF deficiency. Nature *633*, 417–425. https://doi.org/10.1038/s41586-024-07866-3.
103. Leppek, K., Das, R., and Barna, M. (2018). Functional 5′ UTR mRNA structures in eukaryotic translation regulation and how to find them. Nat. Rev. Mol. Cell Biol. *19*, 158–174. https://doi.org/10.1038/nrm.2017.103.
104. Renz, P.F., Valdivia-Francia, F., and Sendoel, A. (2020). Some like it translated: small ORFs in the 5′ UTR. Exp. Cell Res. *396*, 112229. https://doi.org/10.1016/j.yexcr.2020.112229.
105. Silva, J., Fernandes, R., and Romão, L. (2019). Translational Regulation by Upstream Open Reading Frames and Human Diseases. Adv. Exp. Med. Biol. *1157*, 99–116. https://doi.org/10.1007/978-3-030-19966-1_5.
106. Li, G., Wu, J., and Wang, X. (2024). Predicting functional UTR variants by integrating region-specific features. Brief. Bioinform. *25*, bbae248. https://doi.org/10.1093/bib/bbae248.
107. Li, Z., Li, X., Zhou, H., Gaynor, S.M., Selvaraj, M.S., Arapoglou, T., Quick, C., Liu, Y., Chen, H., Sun, R., et al. (2022). A framework for detecting noncoding rare-variant associations of large-scale whole-genome sequencing studies. Nat. Methods *19*, 1599–1611. https://doi.org/10.1038/s41592-022-01640-x.

The American Journal of Human Genetics, Volume *113*

Supplemental information

Genome-wide detection of human 5′ UTR variants that impact protein translation

Matthieu Chaldebas, Khoren Ponsin, Jonathan Bohlen, Clement Conil, Haralambos Mourelatos, Peter D. Stenson, David N. Cooper, Laurent Abel, Jean-Laurent Casanova, Aurélie Cobat, and Peng Zhang

Supplemental Figures

Figure S1: Comparison of WES and WGS coverage on MANE transcript 5' UTR intervals. Distribution of per-sample coverage metrics for 15,680 whole-exome sequencing (WES) and 1,721 whole-genome sequencing (WGS) samples for our in-house database. Each panel displays violin plots showing distribution density, overlaid box plots depicting the median (center line), interquartile range (IQR; box edges), and whiskers extending to 1.5 times the IQR (or the most extreme data point within that range). (a) Median sequencing coverage per sample calculated across 5'UTRs (y-axis on a $\log_{10}$ scale). (b) Distribution of the percentage of the 5'UTR covered at a depth of at least 10X. (c) Distribution of the percentage of the 5'UTR covered at a depth of at least 30X.

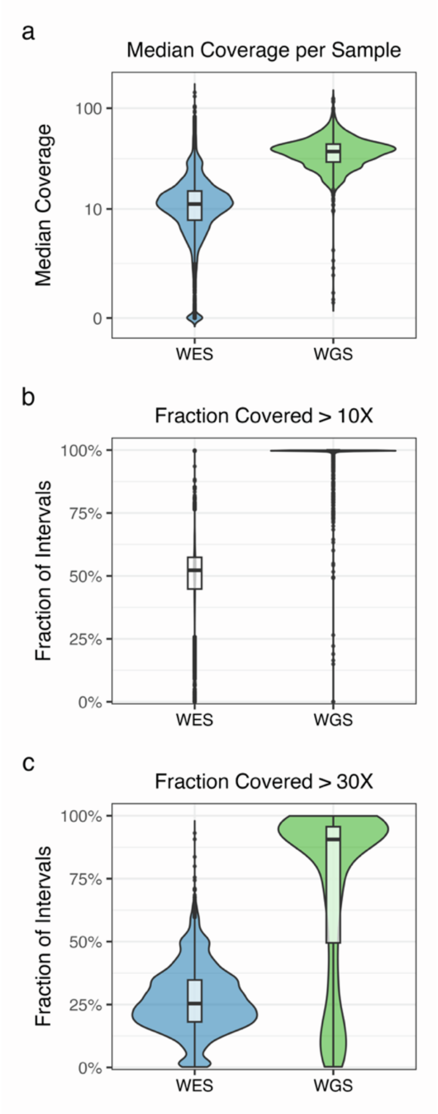

Figure S2: **Scalability analysis of 5ULTRA runtime components.** iMac runtimes were measured on an iMac18.3 equipped with a 4.2 GHz Quad-Core Intel Core i7 processor (4 cores) and 40 GB of RAM. HPC runtimes were measured on a core of an HPC computer node with 25 GB of allocatable memory running Enterprise Linux 9.

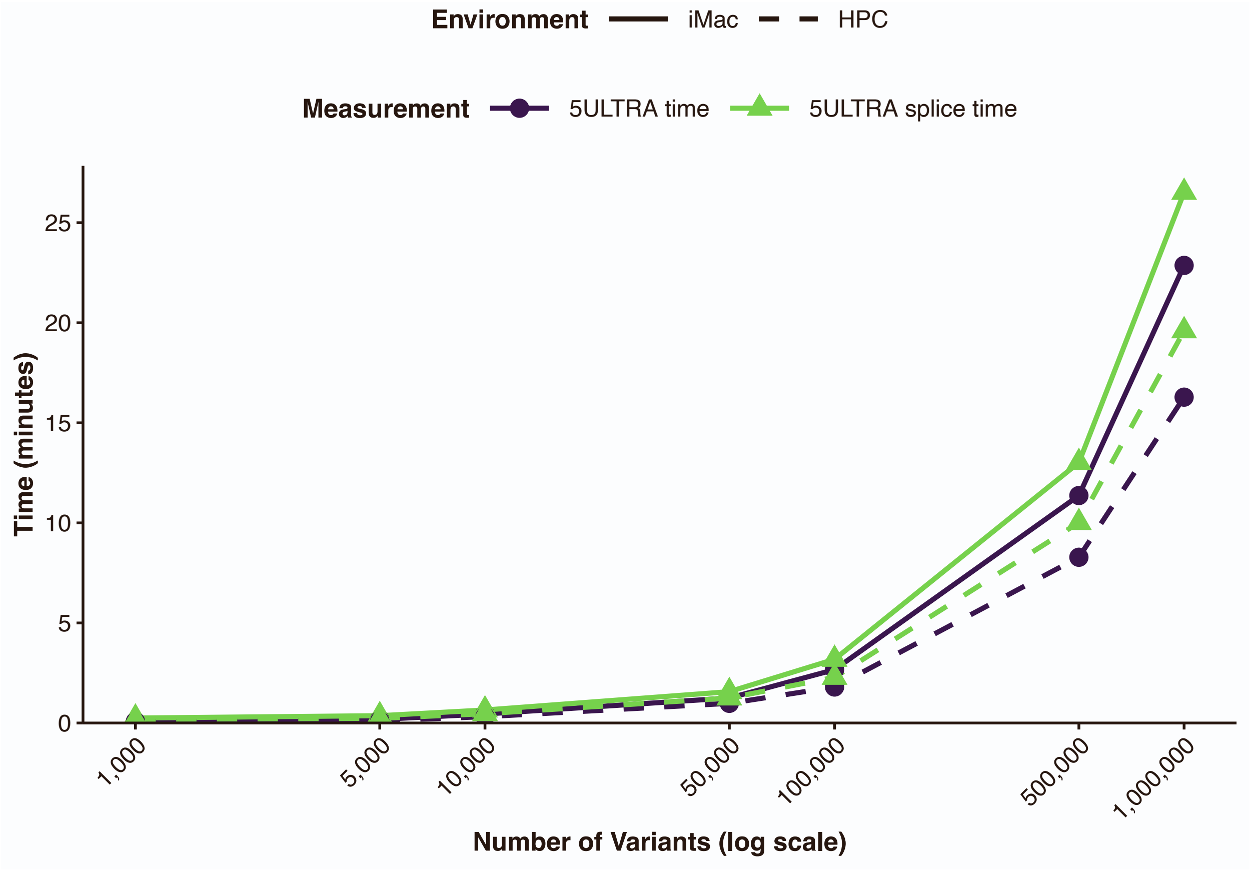

Figure S3: **Allele frequency and conservation by 5'UTR consequence.** (a) MAF distributions across variant categories. (b) PhastCons distributions. (c) PhyloP distributions.

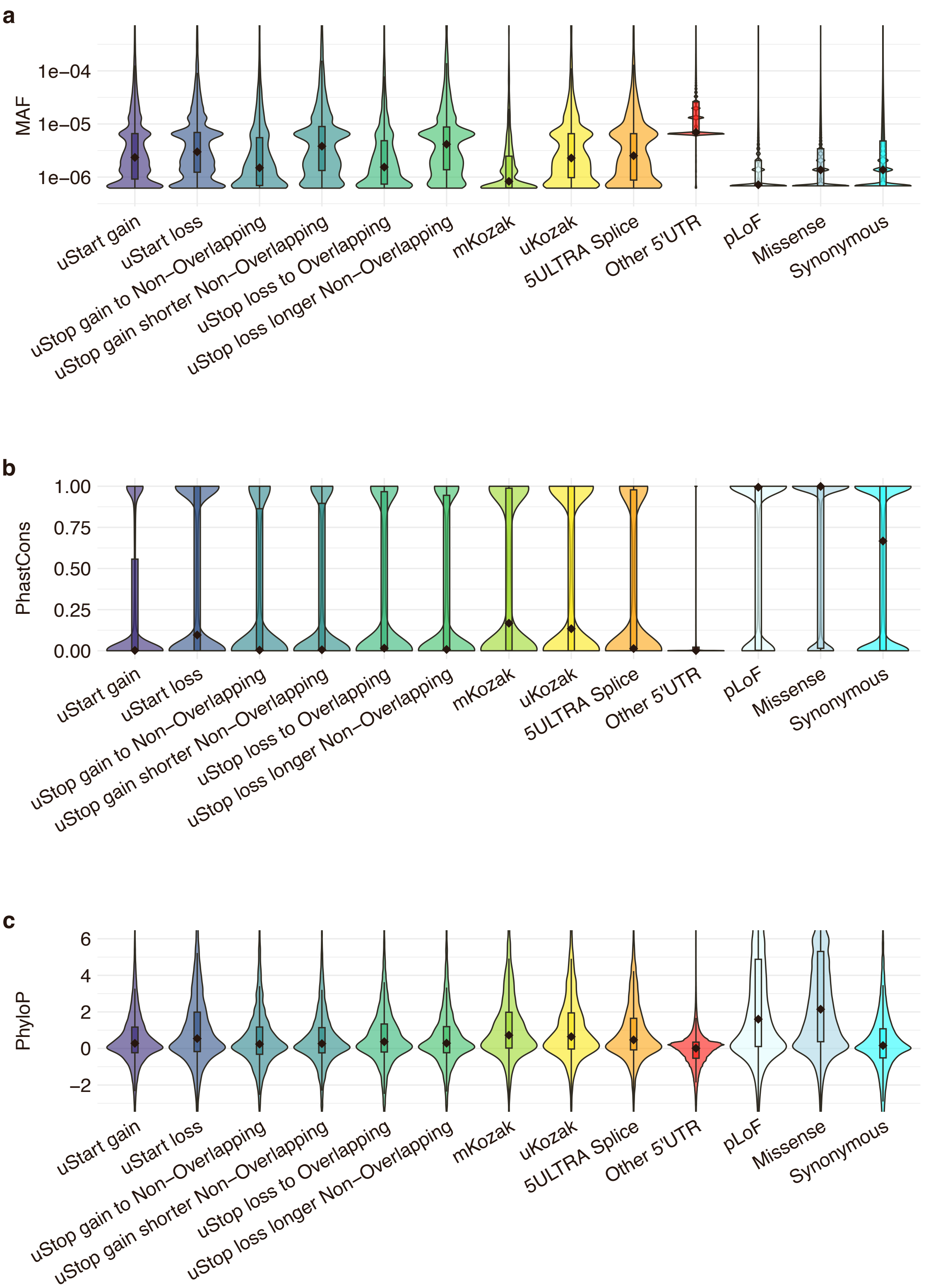

Figure S4: Feature ablation analysis. Relative importance of each feature in the predictive model. Each bar represents the decrease in the mean Area Under the Curve (AUC) from the baseline model (0.98) after the removal of that specific feature. A larger drop in AUC indicates a greater contribution of that feature to the model's overall performance. The analysis was performed using 5-fold cross-validation.

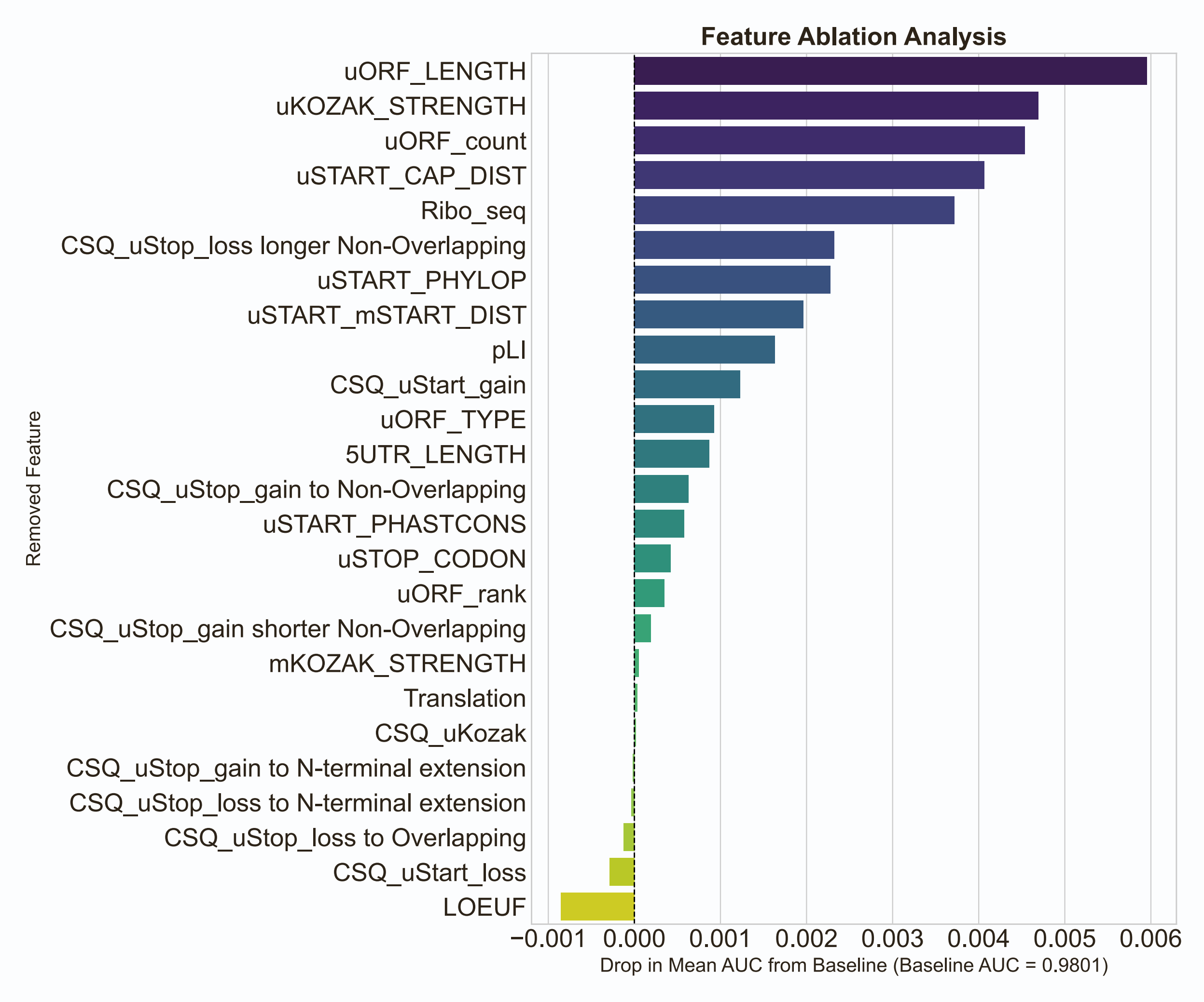

Figure S5: Comprehensive performance evaluation of 5ULTRA and CADD v1.7 on the ClinVar 5'UTR variant dataset. (a) Comprehensive performance metrics at common thresholds. This bar chart compares sensitivity, specificity, accuracy, and Matthews Correlation Coefficient (MCC). (b) Comprehensive performance metrics at common thresholds in the rare variants subset. (c) Precision-Recall (PR) curve for the pathogenic class. The plot shows precision (positive predictive value) versus recall (sensitivity) for 5ULTRA (gold) and CADD (blue). (d) Precision-Recall (PR) curve for the benign class. (e) Decision Curve Analysis (DCA). The plot displays the net benefit of using each model for clinical decision-making across a range of threshold probabilities. (f) Reliability curve comparison. This plot assesses model calibration by binning variants by deciles and plotting it against the observed proportion of pathogenic variants. The dashed line represents perfect calibration. CADD scores were min-max scaled to 0-1.

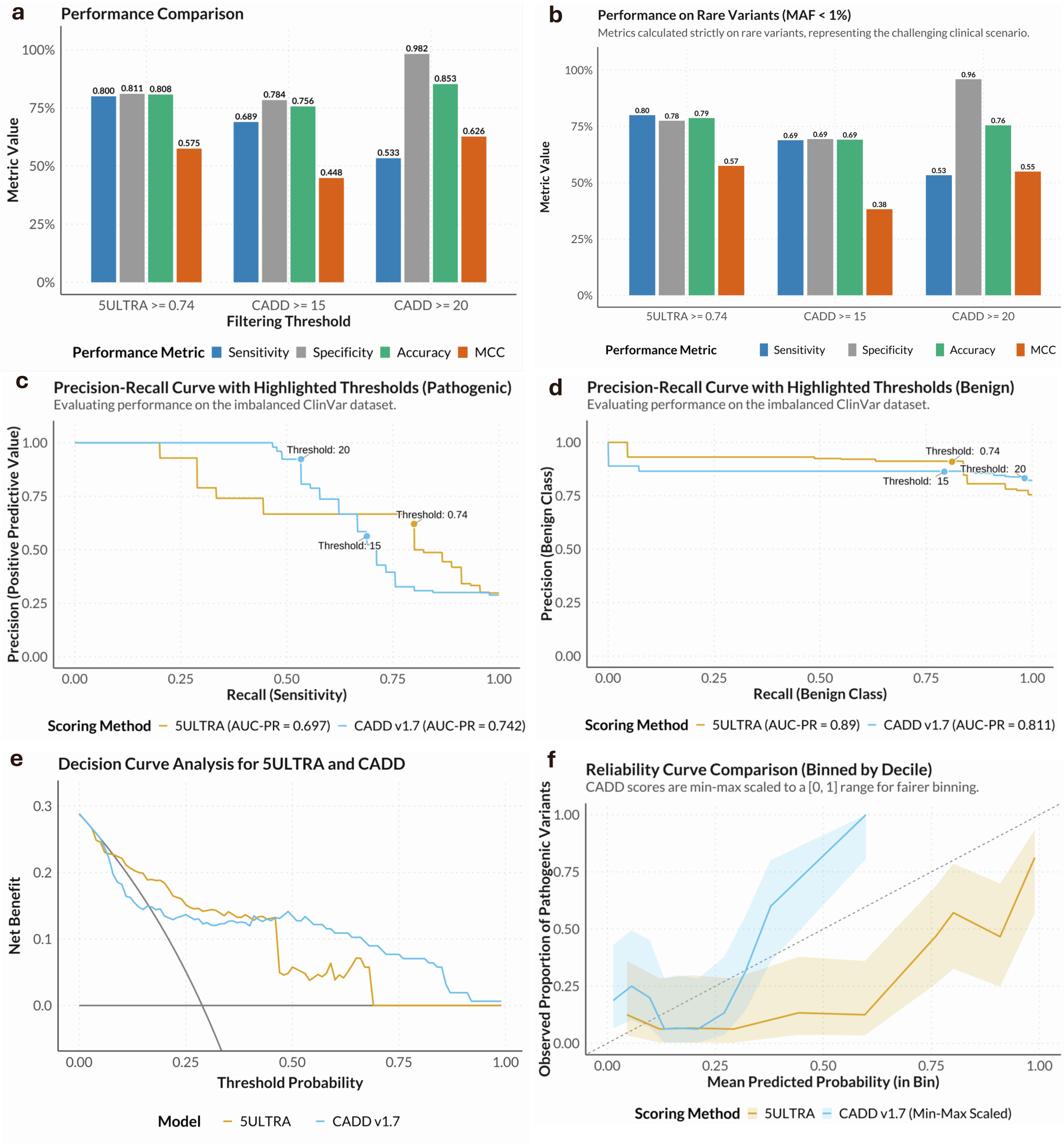
a
Performance Comparison
Metric Value
100%
75%
50%
25%
0%
0.800
0.811
0.808
0.575
0.689
0.784
0.756
0.448
0.533
0.982
0.853
0.626
5ULTRA >= 0.74
CADD >= 15
CADD >= 20
Filtering Threshold
Performance Metric
Sensitivity
Specificity
Accuracy
MCC
b
Performance on Rare Variants (MAF < 1%)
Metrics calculated strictly on rare variants, representing the challenging clinical scenario.
Metric Value
0.80
0.78
0.79
0.57
0.69
0.69
0.69
0.38
0.53
0.96
0.76
0.55
c
Precision-Recall Curve with Highlighted Thresholds (Pathogenic)
Evaluating performance on the imbalanced ClinVar dataset.
Precision (Positive Predictive Value)
Threshold: 20
Threshold: 0.74
Threshold: 15
Recall (Sensitivity)
Scoring Method
5ULTRA (AUC-PR = 0.697)
CADD v1.7 (AUC-PR = 0.742)
d
Precision-Recall Curve with Highlighted Thresholds (Benign)
Evaluating performance on the imbalanced ClinVar dataset.
Precision (Benign Class)
Threshold: 0.74
Threshold: 20
Threshold: 15
Recall (Benign Class)
Scoring Method
5ULTRA (AUC-PR = 0.89)
CADD v1.7 (AUC-PR = 0.811)
e
Decision Curve Analysis for 5ULTRA and CADD
Net Benefit
Threshold Probability
Model
5ULTRA
CADD v1.7
f
Reliability Curve Comparison (Binned by Decile)
CADD scores are min-max scaled to a [0, 1] range for fairer binning.
Observed Proportion of Pathogenic Variants
Mean Predicted Probability (in Bin)
Scoring Method
5ULTRA
CADD v1.7 (Min-Max Scaled)

Figure S6: 5ULTRA and CADD scores correlations with experimental translation effects from MPRA. (a) 5ULTRA vs. Delta MRL: The direction-corrected 5ULTRA score for 38 variants shows a significant and high positive correlation with the measured change in ribosome loading (Delta MRL). The plot shows the linear regression fit (blue line) with its 95% confidence interval. (b) CADD PHRED scores vs. absolute Delta MRL: a total of 1,534 5'UTR variants with a CADD PHRED score show no significant correlation with the measured absolute change in ribosome loading (absolute Delta MRL). The plot shows the linear regression fit (red line) with its 95% confidence interval.

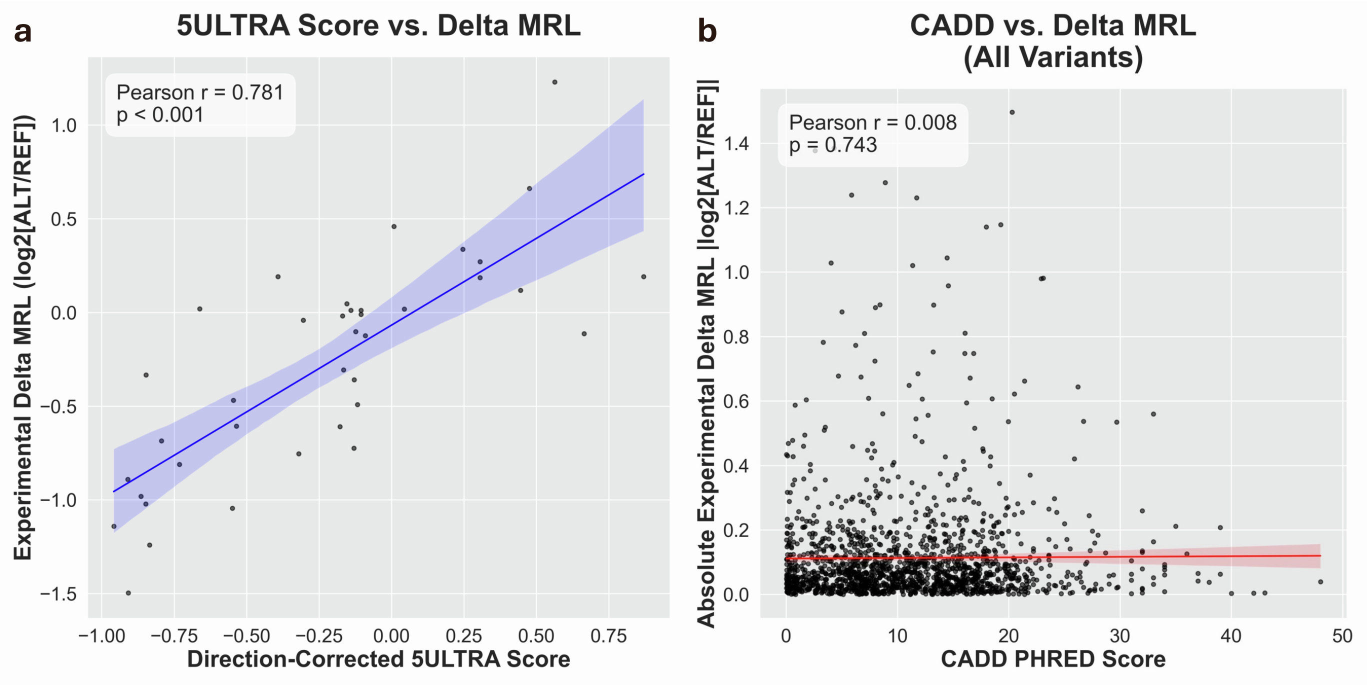

Figure S7: Enrichment in variants with high 5ULTRA scores among CGC Genes. Comparison of proportions of variants located within Cancer Gene Census (CGC) genes.

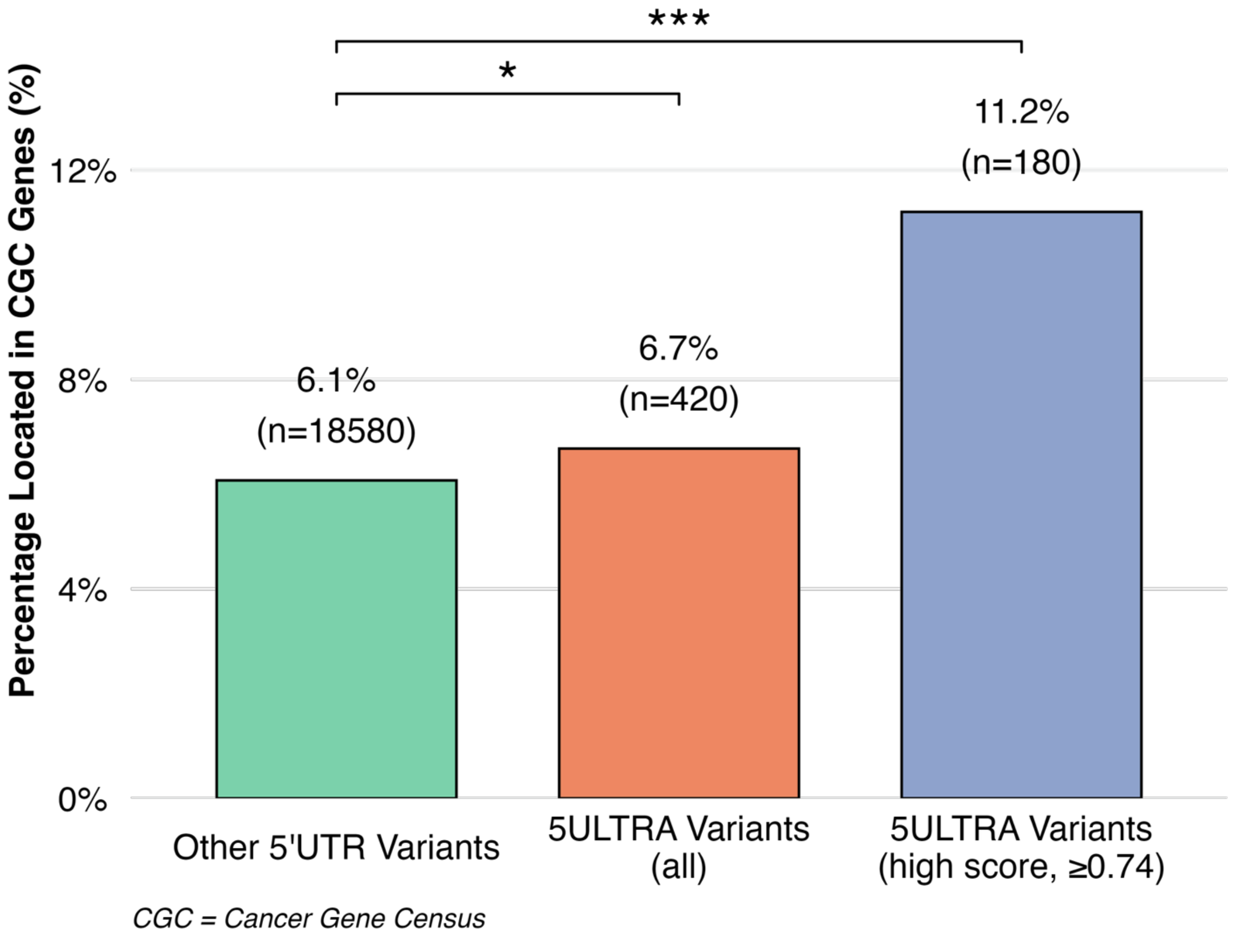

Supplemental Table Legends

Table S1: Accessory genes were defined as the union of 382 protein-coding olfactory receptor (OR) genes (Gene Ontology Molecular Function term = olfactory receptor activity [GO: 0004984]), and 190 dispensable genes.

Table S2: Features used to train the 5ULTRA score random forest model.

Table S3: 36 variants from ClinVar test set detected by 5ULTRA but missed by UTRAnnotator.

Table S4: 420 somatic variants from the COSMIC database, located in GCG genes and detected by 5ULTRA.

Table S5: 25 variants significantly associated with various phenotypes from the GWAS catalog, detected by 5ULTRA.

Supplemental Acknowledgements

H.M. was supported by the Eunice Kennedy Shriver National Institute of Child Health & Human Development of the National Institutes of Health under award number F30HD116571, and a NIGMS/NIH Medical Scientist Training Program grant T32GM152349 to the Weill Cornell/Rockefeller/Sloan Kettering Tri-Institutional MD-PhD Program. J.B. was supported by fellowships from the European Molecular Biology Organization and Marie Skłodowska-Curie Actions postdoctoral fellowship. P.D.S. and D.N.C. acknowledge financial support from Qiagen Inc through a License Agreement with Cardiff University.
